# Development of guidelines for giving community presentations about eating disorders: a Delphi study

**DOI:** 10.1186/s40337-017-0183-x

**Published:** 2017-11-21

**Authors:** Joanna Rachel Doley, Laura Merilyn Hart, Arthur Anthony Stukas, Amy Joanna Morgan, Danielle Lisa Rowlands, Susan Jessica Paxton

**Affiliations:** 1 La Trobe University, Melbourne Campus, Melbourne, Australia; 2 The Butterfly Foundation, Glen Iris, Australia

**Keywords:** Eating disorders, Delphi technique, Mental health literacy, Social stigma, Consumer advocacy

## Abstract

**Background:**

Concerns exist around how to talk about eating disorders (EDs) due to evidence that suggests discussing ED symptoms and behaviours may cause or worsen symptoms in vulnerable people. Using expert consensus, we developed a set of guidelines for giving safe community presentations about EDs.

**Methods:**

Participants with professional ED expertise, and people with lived experience of an ED, were recruited for a Delphi study. *N* = 26 panel members rated 367 statements for both a) inclusion in guidelines, and b) their potential to be helpful (increase knowledge, reduce stigma) or harmful (increase stigma, cause/worsen ED symptoms). After each round of the study, statements were classified as endorsed, re-rate, or not endorsed.

**Results:**

208 statements were endorsed by the panel over three rounds. 13 statements were strongly endorsed in the first round, with both people with lived experience and professionals agreeing it is important for presentations to include information on etiology of EDs and to promote help-seeking. Several statements had a high level of disagreement between those with lived experience and professionals, including the idea that presentations should suggest dieting is likely to result in weight gain.

**Discussion:**

The experts were able to develop consensus on a wide range of issues. Panel members, particularly people with lived experience, were sensitive to aspects of presentations that may be harmful to an audience. The guidelines fill an important gap in the literature and provide guidance to those educating the public about EDs; they should, however, be further evaluated to test their efficacy.

**Electronic supplementary material:**

The online version of this article (10.1186/s40337-017-0183-x) contains supplementary material, which is available to authorized users.

## Plain English summary

There are some concerns that talking about eating disorders in a detailed way (e.g., mentioning low weight, unhealthy weight control behaviours) could be harmful for people who are at risk of eating disorders. However, we know that educating the community about eating disorders is important to reduce stigma and increase help-seeking, so we recruited 26 experts (both professionals and people recovered from an eating disorder) in the field of eating disorders to develop guidelines for how to give safe and effective presentations to the community about eating disorders. Through three rounds of a survey, the experts were able to agree on 208 statements about giving a safe and effective presentation about eating disorders to people in the community. Some areas were not agreed upon by the panel, including giving presentations to children (under 12), and advising people that dieting leads to weight gain. The final results have been developed into a set of guidelines for public use, however they require further evaluation due to the small number of participants.

## Background

Members of the community frequently report stigmatising attitudes towards people with eating disorders (EDs) [1, 2] and low levels of knowledge about these disorders [3]. Stigma and low knowledge about eating disorders (poor mental health literacy) are equally problematic: stigma forms a barrier to treatment seeking [4, 5], and people with poor mental health literacy about EDs may be unaware of evidence based treatments, thus choosing or suggesting potentially ineffective treatments [6, 7]. To reduce stigma and increase mental health literacy, many ED advocacy organisations give presentations to community members about EDs [8–10]. While such presentations are not typically evaluated, similar presentations used in intervention studies show they can increase mental health literacy [11] and reduce stigma [3, 11]. There is a small amount of evidence to suggest, however, that including graphic details about ED behaviours may cause or worsen symptoms in vulnerable individuals [12–14]. Indeed, little research exists on which specific aspects of a community-based presentation could cause harm, and which are likely to be effective in educating the audience and reducing ED stigma. Thus, we aimed to establish a set of guidelines, based on expert consensus, on how to discuss EDs safely in community settings.

### How might talking about EDs cause or worsen symptoms in vulnerable persons?

O’Dea articulated aspects of prevention initiatives that could potentially cause or worsen ED symptoms in young audiences [13]. In particular, O’Dea warned that presentations may introduce young people to awareness of weight/diet concerns, give suggestive information about weight control behaviours/methods (e.g., laxatives, smoking), glamourize EDs (e.g., by including examples of celebrities with EDs), normalize EDs (by making EDs seem more common than they really are), involve transference of poor body image or fear of food from presenter to audience, and introduce a negative focus on food (e.g., by referring to food as ‘good’ or ‘bad’).

Two empirical studies have pointed to the possibility that discussing EDs may have potential dangers; Mann et al. [12] and Carter et al. [15]. Mann and colleagues [12] conducted a prevention program in which two college-aged women (one recovered, the other not recovered) spoke about their lived experience, and EDs more generally, including descriptions of the most severe symptoms. Audience participants were *N* = 788 college-aged women. No significant differences were found between the intervention and control (no intervention) conditions at T1 (three months before the talk). At T2 (four weeks following the talk), however, those in the intervention group reported higher ED symptoms than the control group, although these effects were not apparent at T3 (twelve weeks after the intervention). The intervention group did, however, report that they believed EDs and ED behaviours were more common among students on campus than the control group at both T2 and T3. Mann et al. [12] proposed that the intervention may have normalised EDs, removing the stigma of the behaviours and potentially contributing to the increase in symptoms at T2.

Carter and colleagues’ [15] program was a pilot study of a cognitive behavioural intervention targeting dietary restraint and body dissatisfaction that included information about EDs and their characteristics. Participants were 46 13- and 14- year old girls. Dietary restraint decreased at post-test, but increased at 6-month follow-up to above baseline levels. The reason for this increase is unclear, as the pilot study did not include a control group. In a controlled follow-up study, Stewart et al., (2001) [16] failed to replicate these results; the intervention group improved on outcome variables at post-test, but increased to the same level as the control group at follow-up. These results suggest that the increase in dietary restraint reported by Carter et al. [15] may have been attributable to a ‘natural’ increase in disordered eating throughout adolescence. Despite these two studies being highly influential in shaping how the ED field understands the risk of providing information about ED to community members [17], the evidence of iatrogenesis remains unclear. In addition, two meta-analyses of ED prevention programs conducted by Stice and Shaw [18] and Fingeret, Warren, Cepeda-Benito, and Gleaves [19] found no evidence of iatrogenic effects in prevention programs. Likewise, Becker and colleagues [20] found no evidence of iatrogenic effects using dissonance-based eating disorder prevention efforts which reduce ED symptoms among high-risk groups, and do not result in an increase in symptoms among low-risk groups.

One aspect of community ED presentations has specifically been queried for its potential to cause harm; namely, the use of persons recovered from EDs as presenters. For example, Schwartz, Thomas, Bohan, and Vartanian [21] found that high school students reported more favourable attitudes towards people with EDs (such as ‘girls with eating disorders are very pretty’, and ‘it would be nice to look like the presenter’) when the presenter was introduced as a person recovered from an ED, than when they were introduced as an eating disorder specialist. Heinze, Wertheim, and Kashima [22], however, found no differences between groups on ED risk factors (drive for thinness, dieting intentions, body dissatisfaction) when the presenter of an ED prevention video was introduced as a recovered patient, expert, or peer. Decreases in ED-relevant variables were seen across all groups, with the exception of body dissatisfaction, which was unchanged. Notably, knowledge of ED increased. Qualitative work more relevant to other age groups has also noted that persons with EDs report some negative experiences when reading ED memoirs (e.g., using the memoirs as a ‘guidebook’ with tips on how to maintain their ED [23]).

In sum, the evidence for causing harm through discussing EDs in ‘unsafe’ ways is unclear – while there are reports from people with EDs that particularly graphic descriptions of symptoms may cause negative effects for them, it is unclear how *much* information would produce negative consequences. In non-ED samples, it is unclear whether presentations are causing genuine increases in ED symptoms. It appears that a nuanced approach to the problem of how to talk about EDs is required; although an uninformed attempt to discuss EDs may cause harm, there are notable benefits to discussing EDs safely [3, 11]. Recommendations for how to talk about EDs in the media [24, 25] and how to assist in school prevention of ED [26, 27] have been established; however, there are no specific evidence-based guidelines for discussing EDs with community groups where the intention is not to prevent EDs, but rather to increase mental health literacy and to reduce stigma.

### The present study

Identifying aspects of community ED presentations that may have benefit or may cause harm, is not feasible for two reasons: 1) it is unethical to expose individuals to aspects of an ED presentation that may induce ED symptoms or behaviours; and 2) there are too many aspects of a presentation to vary in an experimental context (e.g., multiple symptoms, risk factors, presentation modes). These are circumstances under which the use of the Delphi method is most appropriate [28]. The Delphi method facilitates expert consensus to answer an overarching research question [28]. It has frequently been used in mental health research, including in the context of eating disorders, for instance in the development of Mental Health First Aid Guidelines for EDs [29]. In this research, we used the expertise of both professionals within the ED field and people with lived experience of ED, to make informed recommendations about how to give an effective and safe presentation about EDs to the community.

## Method

### Participants

People over 18 years of age from two broad categories were eligible to participate in the study: professionals in the field of EDs (e.g. clinicians, researchers, and community educators), and people with lived experience of an ED who work or volunteer in an advocacy role (e.g., bloggers, speakers). Participants were identified in one of several ways: membership in a relevant body (e.g., Australian and New Zealand Academy for Eating Disorders), association with a relevant group (e.g., working with a non-profit ED organisation), through public profile (e.g., social media presence) or through recommendations of others in the ED field. Participants were invited directly via email or via advertisement through relevant organisations. Participants with a history of an ED were required to have been recovered for a minimum of 2 years without any significant ED thoughts or behaviours (self-identified by agreeing to take part in the study after reading the participant information statement).

**Fig. 1:**
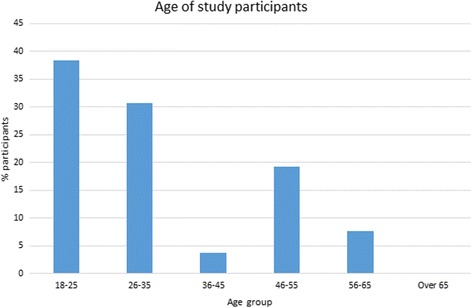
Age groups of expert panel

**Fig. 2:**
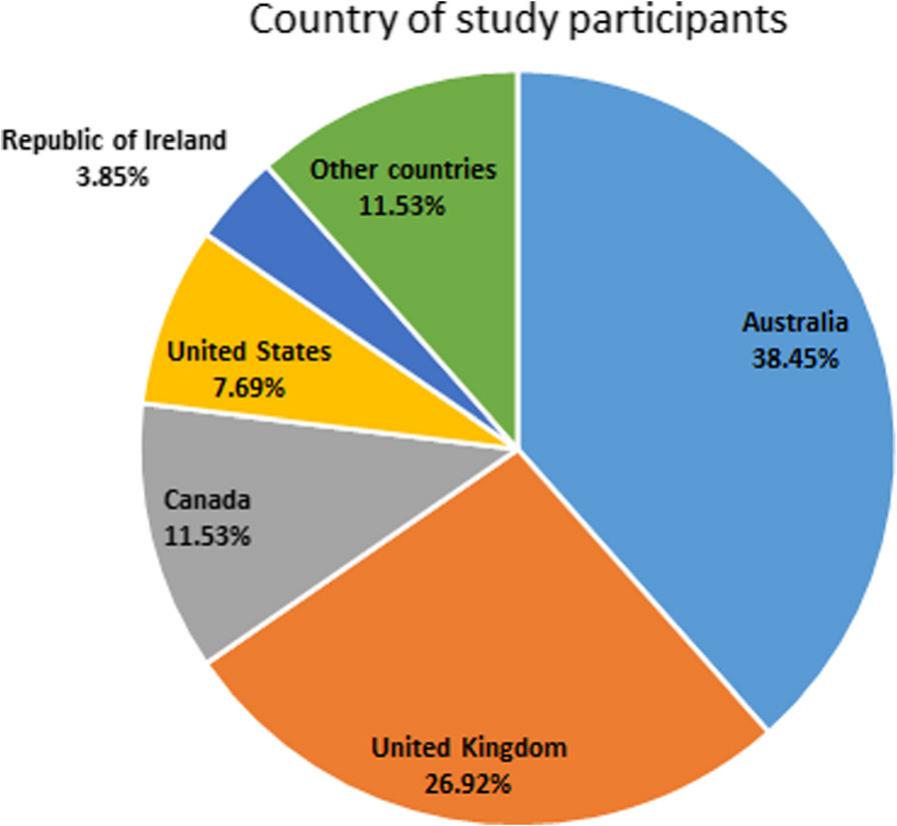
Country of expert panel members

A total of *N* = 26 panel members consented to participate in the study (*n* = 16 lived experience, *n* = 10 professionals). The panel was almost all female (*n* = 25), with no male participants, and one participant indicating “other” as their gender (but not specifying their gender identification). Of participants, most were aged 18–25 or 26–35 (see Fig. 1), with younger persons more likely to complete all three rounds than older persons – at round 2, 23.44% of the panel were over 46 years of age, while at round 3, 7.6% of the panel was over 46 years of age. A range of countries were represented in the panel (see Fig. 2), with the largest number of participants from Australia. Panel members generally indicated that they were experienced in the eating disorders field, with *M* = 9.92 years of experience (*SD* = 11.10). As this was positively skewed, we examined the median, which was $\overline{x}=$7.00 years.

### Measures

The first author (JD) completed a systematic search of both the academic and non-academic literature (e.g., peer-reviewed articles, websites of ED advocacy organisations) to collect statements advising presenters about how to talk about eating disorders in the community. Four databases were searched (Google US, UK, and Australia; Google Scholar; PsycINFO; Web of Science) using the term *eating disorder* with each of the following terms: *talk, speaker guidelines, prevention guidelines, stigma, community education, what not to say*. The first 50 results for each search engine were screened. Any potentially relevant links to other websites from these results were followed. Any unique statement concerning how to talk about eating disorders was extracted and entered into a spreadsheet, irrespective of whether or not the authors believed the concept would be helpful or harmful.

Gleaned information was re-written (by author JD) into statements providing guidance about how to give a community presentation about EDs, while attempting to retain the original meaning of the source. For instance, “Share concrete ways that friends and loved ones helped you or could have helped you so audience members learn ways they can be there for their loved ones” was re-written as “lived experience speakers should talk about what their loved ones did to help them through recovery”. To organize statements, broad categories were developed based on stages of giving a presentation (e.g., planning a presentation, during the presentation). Sub-categories were then developed to structure the statements in each category (e.g., sub-categories for content of a presentation: multimedia/images, messages/language, signs/symptoms, causes/risk factors, recovery). The statements and categories were refined for clarity by a working group (including authors JD, LH, AM, DR, and SP).

#### Rating scales

Two types of rating scales were used for each statement. The first measured the importance of an item being included in guidelines. Responses ranged from 1 = Essential, 2 = Important, 3 = Don’t know/depends, 4 = Unimportant, to 5 = Should not be included, in line with previous studies [28]. The second assessed whether a statement should be included or excluded based on its potential to be helpful or harmful. We defined helpful as anything that would increase knowledge, reduce stigma, or reduce ED symptoms. Conversely, we defined harmful as anything that would increase stigma or ED symptoms. It should be noted that reduction in ED symptoms did not refer to a prevention context; rather, we are acknowledging that this is the inverse of harm (i.e., increasing ED symptoms), and not typically an intended consequence of community presentations. Responses ranged from 1 = Very helpful, 2 = Helpful, 3 = Don’t know/depends, 4 = Harmful, to 5 = Very harmful.

Statements were also divided by the age of the audience in a presentation. Each statement was rated for its appropriateness and helpfulness/potential for harm in both adolescent (12–17 years) and adult (18+ years) audiences. Several additional statements included scales for appropriateness in child (under 12 years) audiences, for speakers with lived experience, and an audience of professionals (e.g., doctors).

### Procedure

Participants provided informed consent to take part in the Delphi study, which was approved by the Institution Review Board (UHEC HRE-15-062). Participants completed the questionnaire online through Qualtrics.com. Responses were kept confidential; the email address of each participant was, however, visible to the researchers in order to contact them with personalized feedback containing their own responses, and the responses of the panel in group average format.

The first questionnaire took approximately one hour to complete. Participants rated each statement on its relevant scales, and were invited to provide qualitative feedback at the end of each sub-category in order to suggest any new ideas or changes to statements, or any experiences they believed were relevant (e.g., observing whether presentations to adolescent mixed-gender audiences were effective). Once panellists had lodged their responses, data were analysed. Each statement was classified as either ‘Endorsed’ (meaning it would appear in the guidelines and not rated in subsequent rounds and), ‘Re-rate’ (meaning it would appear in the next round for panel members to re-rate), or ‘Not endorsed’ (meaning it would not appear in subsequent rounds, and not be included in the guidelines), according to the criteria in Table 1. Delphi cut-off criteria depends partly on the statement being evaluated and partly on the number of groups (i.e., diversity of panels and experience [28]). This study used cut-offs similar to other Delphi studies in the field of community mental health and more specifically, EDs, that have included both consumer and professional groups [30, 31].

**Table 1:** Criteria for classifying questionnaire statements

	Inclusion in guidelines		Helpful/harmful
Endorsed	At least 80% of both professionals AND people with lived experience rate the item “essential” or “important”	AND	At least 80% of both professionals AND people with lived experience rate the item as “very helpful” or “somewhat helpful” (or in the case of items we wished to highlight as harmful in the guidelines, “very harmful” or “unhelpful”.)
Re-rate	70–79% of both professionals AND people with lived experience rate the item “essential” or “important”; OR >80% of either professionals OR people with lived experience rate the item “essential” or “important”	AND	70–79% of both professionals AND people with lived experience rate the item “very helpful” or “somewhat helpful” (or in the case of items we wished to highlight as harmful in the guidelines, “very harmful” or “somewhat harmful”); OR >80% of either professionals OR people with lived experience rate the item “very helpful” or “somewhat helpful” (or in the case of items we wished to highlight as harmful in the guidelines, “very harmful” or “somewhat harmful”).
Not endorsed	<70% of both professionals AND people with lived experience rate the item “essential” or “important”	AND/OR	<70% of both professionals AND people with lived experience rate the item “very helpful” or “somewhat helpful” (or in the case of items we wished to highlight as harmful in the guidelines, “very harmful” or “somewhat harmful”).

After categorising the statements from the first round, the working group examined the qualitative feedback and developed new statements for entry into a second round questionnaire. Any novel ideas suggested by the panellists in their feedback were drafted into new questionnaire statements (e.g., some participants did not feel a speaker needed to be endorsed by an ED organisation, but felt speakers should have adequate mental health support, so this was clarified in subsequent rounds). Additionally, several statements that elicited confusion among the panel, due to negative wording for example, were re-written to simplify for re-rating.

The data analysis process was repeated for a second and third round, with the option for panelists to provide qualitative feedback removed in the second round, so no new items entered into the third. The second round questionnaire took approximately 20 min to complete, and the third round took approximately 10 min to complete. Statements without consensus by the third round were categorised as ‘Not endorsed’.

#### Attrition

A number of panel members did not complete the second (*n* = 9) or third (*n* = 13) rounds of the study. We wanted to test whether this attrition had a marked effect on the study results, so we compared the ratings of completers (those who completed all three rounds) with those of non-completers (those who only completed the first or first and second round) at baseline. We selected a random sample of round one items to analyse, with item numbers randomly generated using SPSS. Ten independent samples t-tests were used to compare groups.

#### Guideline document development

Once all data were gathered, the first author wrote the endorsed statements into prose. For example, the questionnaire statement for adolescent audiences “Authors should be aware that females and males may feel uncomfortable discussing body image/eating disorders in front of the opposite gender” became the guideline “It may be helpful when presenting to an adolescent audience, to know that adolescent females and males may feel uncomfortable discussing body image/eating disorders in front of the opposite gender.” This draft was presented to the working group, who met in person to edit for clarity, spelling, grammar, and consistency. Throughout this drafting process the working group attempted to retain the original wording of the statements as closely as possible and to ensure the endorsed meaning of the statements was not altered in any way. The guidelines document (see Additional file 1) was then forwarded to all panellists who were asked to provide feedback on whether the guidelines accurately captured their responses.

## Results

### Literature search

The majority of statements were derived from websites of major ED organisations involved in advocacy and support (e.g., National Eating Disorders Information Centre [32], http://www.nedic.ca/). A smaller number of statements were derived from academic articles (e.g., Ferrari et al., [33]; Mond, [34]). A total of 203 statements were derived from the original search and 179 unique statements were finalised by the working group and presented to the panellists in the first round questionnaire.

### Statements

Across the three rounds, the panel was presented with 192 unique statements, but rated a number of these separately for different audiences, such that there were 367 in total. Panellists rated 179 statements for adults, 175 statements for adolescents, 6 statements for children under 12 years, 6 statements for speakers, and 1 statement for professionals. Panellists endorsed 208 statements (56.68%). See Table 2 for details of the statements, and Additional file 1 for a detailed breakdown of results. Panel attrition was high; 65% and 50% of the original panel members took part in rounds two and three, respectively (see Table 3). As noted, we explored whether attrition had an impact on the findings by comparing the differences between completers’ and non-completers’ ratings in round 1 on ten randomly selected items. From the randomly selected items, we found differences between non-completers and completers on only one of ten items (*p* = .01), indicating that the final results are not likely to be strongly affected by panel attrition across rounds. The single difference was on the item “Authors SHOULD... review presentations and materials for ambiguity and risk of harm on a regular basis” with non-completers endorsing reviewing presentations for ambiguity and risk of harm as more helpful than completers (although still tending to endorse it as helpful). See Additional file 2 for a full description of results.

**Table 2:** Statement ratings and number of statements presented to participants for rating in each round

	Round 1	Round 2	Round 3
Endorsed	102	78	26
Re-rate	132	61	N/A
Not endorsed	106	20	35
New Items	0	27	0
Total	340	159	61

**Table 3:** Number of panel members who completed each round of the study

	Round 1	Round 2 (% retained from original panel)	Round 3 (% retained from original panel)
Lived experience	16	10 (62.5%)	8 (50.0%)
Professionals	10	7 (70.0%)	5 (50.0%)
Total	26	17 (65.4%)	13 (50.0%)

#### Unanimous recommendations

Within the first round, 13 statements, shown in Table 4, were strongly endorsed (i.e., 100% of participants agreed that they were Essential or Important for inclusion in the guidelines, and Very Helpful or Helpful in reducing stigma, increasing knowledge, or reducing ED symptoms) by both professionals and people with lived experience. The panel unanimously agreed on statements that promoted an inclusive environment in which help-seeking is encouraged. The panel agreed on the importance of presenters as good role models and agreed that presentations should emphasise help-seeking and asking for support. Additionally, they agreed that presenters should teach the biopsychosocial model of EDs, and that people from all backgrounds and demographics can develop an ED.

**Table 4:** Strongly endorsed items rated as “Essential” or “Important” AND “Very helpful” or “Helpful” by 100% of participants

Statement and questionnaire category	Agreement across all scales (%)
Presentation format	
Authors and presenters should... be good role models and advocate for a safe and respectful environment free from discrimination (12–17)	100
Authors and presenters should... be good role models and advocate for a safe and respectful environment free from discrimination (18+)	100
Presentation content - Signs and symptoms	
Presentations should... include warning signs of eating disorders (18+)	100
Presentation content - Causes/risk factors	
Authors and presenters should... explain that a combination of biological, psychological and sociocultural factors contribute to the development of eating disorders (12–17)	100
Authors and presenters should... explain that a combination of biological, psychological and sociocultural factors contribute to the development of eating disorders (18+)	100
Authors and presenters should... explain that eating disorders affect people regardless of their gender, race, ethnicity, socioeconomic status, or sexual orientation (12–17)	100
Authors and presenters should... explain that eating disorders affect people regardless of their gender, race, ethnicity, socioeconomic status, or sexual orientation (18+)	100
Presentation content - Recovery	
Authors and presenters should... tell the audience that it is courageous and necessary to ask for help and support during recovery from an eating disorder (12–17)	100
Authors and presenters should... tell the audience that it is courageous and necessary to ask for help and support during recovery from an eating disorder (18+)	100
Presentation content - Seeking help	
Presentations should... include information about eating disorder support services (12–17)	100
Presentations should... include information about eating disorder support services (18+)	100
Presentations should... normalise help-seeking (12–17)	100
Presentations should... normalise help-seeking (18+)	100

#### Differences between professionals and people with lived experience

In the first round, a number of statements, shown in Table 5, had a high level of discrepancy between professionals and those with lived experience (i.e., one group’s rating placed the item in the ‘endorsed’ category and the other group’s rating placed the item in the ‘not endorsed’ category). Some statements went on to be endorsed by both groups in subsequent rounds, although some failed to be endorsed by both groups. One of the most interesting findings was the discrepancy between the professional and lived experience groups for items concerning dieting and body weight. In particular, while professionals endorsed explaining the effects of dieting on body weight regulation, and that dieting often results in weight *gain*, those with lived experience rejected these items. One panel member who included a qualitative response to this item, stated;

If we're trying to prevent eating disorders by explaining that disordered eating behavior might make you gain weight, there's something backward in our reasoning. Let's try to stop using weight gain as the monster under the bed that we should avoid at all costs—especially because many people, in recovery or otherwise, will actually become healthier as they gain weight. (*Participant 2, lived experience).*

**Table 5:** Statements with notable discrepancies between professionals’ and people with lived experience’s ratings

	Professionals		Lived Experience	
	Inclusion (%)	Helpful (%)	Inclusion	Helpful (%)
Lived experience speakers – Do no harm				
LE Speakers should... be prepared to address common myths about eating disorders (18+)^a^	45.45	60.00	93.33	93.33
Content – Multimedia and images				
Authors should… consider showing videos that promote body image media literacy (12–17)	81.82	90.00	57.14	64.29
Content – physical signs/symptoms				
Authors and presenters should… explain the effects of dieting on body weight regulation (12–17)	90.91	100.00	57.14	57.14
Presentations should… explain that dieting often causes weight gain (12–17)	90.91	100	50.00	42.86
Presentations should… explain that dieting often causes weight gain (18+)	90.91	100	53.33	46.67
Content – discussing recovery				
Authors and presenters should... explain that most people who recover from an eating disorder do so with the support of both trained professionals, family and/or friends, rather than on their own. (12–17)^a^	81.82	80.00	53.85	53.85
Authors and presenters should... explain that most people who recover from an eating disorder do so with the support of both trained professionals, family and/or friends, rather than on their own. (18+)^a^	81.82	80.00	57.14	57.14

^a^endorsed in round 2

#### Rejected recommendations

In the first round, items shown in Table 6 were strongly rejected (<40% endorsement across all scales by both groups). Statements about emphasising, or de-emphasising, one particular risk factor over others (e.g., biological factors over environmental factors in etiology) were strongly rejected by the panel. In accordance with the current evidence base and knowledge of how EDs develop [35], there was unanimous agreement that presenters should teach the biopsychosocial model of EDs.

**Table 6:** Statements strongly rejected by both professionals and people with lived experience

	Professionals		Lived Experience	
	Inclusion (%)	Helpful (%)	Inclusion (%)	Helpful (%)
Format of a presentation				
Authors should... present to single sex groups (i.e. female-only or male-only audiences (12–17)	9.09	18.18	21.43	28.57
Authors should... present to single sex groups (i.e. female-only or male-only audiences (18+)	0.00	9.09	20.00	20.00
Presentations should not... include media reports, drama presentations or case studies about eating disorders, as they may trivialise or glamorise the illness (12–17)	18.18	18.18	21.43	35.71
Presentations should not... include media reports, drama presentations or case studies about eating disorders, as they may trivialise or glamorise the illness (18+)	18.18	18.18	20.00	33.33
Presentations should not... invite speakers with lived experience who are peers known to the audience (12–17)	36.36	10.00	28.57	21.43
Presentations should not... invite speakers with lived experience who are peers known to the audience (18+)	27.27	10.00	26.67	20.00
Lived experience speakers – Do no harm				
LE speakers should not... make reference to specific treatment facilities or providers (12–17)	36.36	20.00	21.43	28.57
LE speakers should not... make reference to specific treatment facilities or providers (18+)	36.36	20.00	20.00	20.00
Presentation content – Multimedia and images				
Presentations should not... mention pro-eating disorder websites (18+)	27.27	10.00	26.67	26.67
Presentations should not... include movies depicting persons with eating disorders (12–17)	27.27	10.00	35.71	35.71
Presentations should not... include movies depicting persons with eating disorders (18+)	18.18	10.00	33.33	26.67
Presentation content – Risk factors/causes				
Authors and presenters should not… describe eating disorders as caused by biogenetic/genetic factors because this may reinforce the idea that an eating disorder is an unchangeable characteristic of a person. (12–17)	20.00	22.22	30.77	23.08
Authors and presenters should not… describe eating disorders as caused by biogenetic/genetic factors because this may reinforce the idea that an eating disorder is an unchangeable characteristic of a person. (18+)	20.00	22.22	21.43	21.43
Presentations should not… suggest that psychosocial factors are more controllable than biogenetic factors in the development of an eating disorder (12–17)	30.00	22.22	38.46	30.77
Presentations should not… suggest that psychosocial factors are more controllable than biogenetic factors in the development of an eating disorder (18+)	30.00	25.00	35.71	28.57
Presentations should... describe eating disorders as caused by biogenetic risk factors because this may reduce personal blame for the development of an eating disorder. (12–17)	30.00	33.33	30.77	30.77
Presentations should... describe eating disorders as caused by biogenetic risk factors because this may reduce personal blame for the development of an eating disorder. (18+)	30.00	33.33	35.71	35.71
Authors and presenters should…explain that holding people responsible for their eating disorder behaviour may assist people in their efforts to recover (12–17)	30.00	33.33	15.38	15.38
Authors and presenters should…explain that holding people responsible for their eating disorder behaviour may assist people in their efforts to recover (18+)	30.00	33.33	21.43	21.43
Presentation content - Recovery				
Authors and presenters should not… use stories of people who fought their illness alone (12–17)	36.36	30.00	15.38	15.38
Authors and presenters should not… use stories of people who fought their illness alone (18+)	18.18	11.11	0.00	7.69
Presentations should not... reference specific treatment facilities or providers (12–17)	9.09	30.00	7.69	0.00
Presentations should not... reference specific treatment facilities or providers (18+)	9.09	30.00	7.69	0.00
Special populations – Children under 12 years				
Presentations about eating disorders should not... be given until high school (children aged 12 and up)	27.27	30.00	14.29	7.14
Special populations – athletes				
Authors should… be aware that information about the causes, symptoms, and detrimental effects of eating disorders should only be presented to persons who care for people at risk (e.g. parents, staff, coaches) (12–17)	27.27	20.00	15.38	15.38
Authors should… be aware that information about the causes, symptoms, and detrimental effects of eating disorders should only be presented to persons who care for people at risk (e.g. parents, staff, coaches)(18+)	18.18	20.00	21.43	21.43

The most common statements strongly rejected by panel members were those that advocated excluding certain types of media; for instance, panel members strongly rejected items that suggested presentations should not include media, case studies, or movies about people with EDs. Additionally, panel members strongly rejected items that suggested that pro-ED websites should not be mentioned in presentations, instead advocating that presentations should not reference specific pro-ED websites by name as in the following comment:

If mentioning pro-ana sites ensure it is only briefly and not in detail or name any particular sites. For example, "during my struggles I did visit damaging sites which were very dangerous places". (*Participant 20, lived experience)*

#### Items without clear consensus

Some topics (i.e., several items covering one general idea) did not have a clear consensus by round three. For instance, some of our panel members supported discussing EDs with children under 12, while others suggested that presentations to children under 12 years should only focus on prevention and not mention EDs. Therefore, this topic appeared to be an area of some disagreement between panel members. One panel member pointed out that young children are increasingly aware of EDs:

Children younger than 12 are becoming more aware of eating disorders, it is becoming the norm for younger children to be acting like teenagers. My fear is that if we do not discuss eating disorders and the dangers and help children to know how to seek help we will increase the occurrence. (*Participant 5, lived experience)*

However, another highlighted the potential difficulties of discussing such information:

I think that with young children, presentations should be given about self-esteem and body image and talk vaguely about if they are having troubles with food or their bodies, rather than talking about eating disorders specifically. It should be aimed at encouraging children to talk to someone if they feel bad about their bodies or are not eating properly - although the latter may give children ideas about not eating properly. It's a very tricky subject so maybe just talk about bodies and self-esteem, and talk about it is (sic) a body positive way to encourage good self-esteem and good body image. (*Participant 17, professional).*

As a clear consensus could not be reached, our guidelines included information for those wishing to give presentations to young children that directed them to evidence-based prevention programs.

## Discussion

The aim of our study was to develop a set of guidelines for how to give a community presentation about EDs that was *helpful* (i.e., reduced stigma, increased knowledge, or decreased ED symptoms) and did not cause *harm* (i.e., increase stigma or ED symptoms), by using expert consensus. The guidelines provide recommendations on the format and content (including media, language, and advice for presenters) of ED presentations to adolescent and adult audiences. Although some of the items endorsed reflect common sense principles, like including help-seeking information for the audience and encouraging help-seeking, this is the first time such work has been systematically examined using expert consensus for EDs.

Unanimously agreed-upon statements included ideas or information supported by peer-reviewed literature. For instance, the panel unanimously agreed that presenters should be good role models for their audience. This finding mirrors concerns in O’Dea’s original paper discussing potential problems with school ED prevention initiatives, that teachers or presenters with their own body dissatisfaction or weight bias could impact the quality of the message or transfer poor body image perceptions to the audience [13]. The way the presenter acts both before and following a presentation may affect the credibility of a message – for instance, if a teacher who gives a presentation fails to speak against appearance-based discrimination in their classroom, the message may be seen as inauthentic. Thus, presenters have an important, ongoing, responsibility to their audience. Another statement on which there was unanimous agreement was that authors and presenters should teach the biopsychosocial model of EDs, which is in accordance with the current evidence base and knowledge of how EDs develop [35]. Likewise, the panel unanimously agreed that presentations should teach the audience that EDs affect people regardless of their demographic. While EDs have been traditionally thought of as affecting young, white, heterosexual women, recent literature shows less disparity in prevalence across demographics than previously thought (e.g., by gender, sexuality, age, or ethnicity; [36–38]).

Statements with discrepancies between professionals and those with lived experience were generally those that were evidence-based but had issues highlighted by the lived experience group that were unexamined in the literature. For instance, while it has been a common public health strategy and ED/obesity prevention recommendation to discourage dieting due to its effect on body weight [39, 40], doing so may reinforce negative weight bias. The lived experience group may have been more sensitive than professionals to content that they believed may cause feelings of body dissatisfaction in audience members. Both professionals and those with lived experience supported explaining other harmful effects of dieting that were unrelated to weight loss. Future quantitative research could investigate whether explaining that ‘dieting leads to weight gain’ is harmful (e.g., by examining its effects on weight stigma, self-stigma, and body dissatisfaction).

Statements that the panel rejected were often statements that were inflexible (e.g., Presentations should not... include movies depicting persons with eating disorders.). Some participants noted that some of the strategies given could be used in safer ways – for instance, instead of not mentioning pro-ED websites, they could be spoken about more generally. Likewise, using a certain type of media (e.g., a video about someone with an ED) will not likely be harmful in itself if the content is safe. It is clear that authors and presenters should be thoughtful in choosing content for their presentation, and that they should be aware of their important responsibility to their audience.

### Limitations and strengths

The high attrition in our study may mean that we have less reliable results in the later rounds. However, when we compared responses of completers and non-completers to a random sample of round one statements, we found only one statistically significant difference, indicating that attrition was unlikely to have a marked impact on our findings. Another limitation relates to the demographic characteristics of our panel. No panel members were male, the sample was generally quite young, and more people with lived experience participated than professionals. Our recruitment attempted to cover a broad range of people to join the panel; however it appeared that particular groups were less likely to respond or to complete the study. Thus, our sample may have responded differently than one with men, more professionals, or a larger range of ages. Additionally, while we approached potential panel members who were relatively well-known or credentialed in the ED field, the study was also open to anyone who identified themselves as an ED professional. Thus, we cannot be certain of the quality of expertise in the panel. We also did not gather data on the specific professions of participants; as such, we do not know whether some professions were over or underrepresented. Likewise, those with lived experience were self-identified as recovered, and the definition of recovery can vary depending on the individual. Our inclusion criteria used a specific definition of recovery which may mitigate this limitation somewhat.

The length of the questionnaire may have resulted in lower participation and greater attrition, but the detail of the survey is a strength of this research. We were able to produce a set of guidelines that offer information tailored for presentations to specific age groups, and highlight several aspects of presentations that might be harmful as a caution, as well as things that are likely to be helpful. The inclusion of qualitative data allowed us to interpret some of the responses more clearly than if only quantitative responses were obtained. Additionally, the majority of items in the questionnaire reached consensus in the first round, so not all of the study was impacted by attrition. At present, the guidelines are best considered somewhat preliminary due to the narrow range of demographic characteristics of participants and the high degree of attrition in later rounds. Future research should evaluate the effectiveness of presentations that follow the guidelines, as opposed to those that do not, to ensure that this research adequately translates into presentations producing real reductions in stigma, increases in ED mental health literacy, and no iatrogenic effects.

## Conclusions

This Delphi research developed expert consensus on how to give an effective and safe presentation about EDs to people in the community. Panel members endorsed a range of statements covering all aspects of a presentation, designed for either adolescent or adult audiences. These guidelines are the first of their kind to be produced and will help guide public health approaches to ED education and awareness.

## Additional files

Additional file 1: Complete list of items endorsed or not endorsed by questionnaire round. **Table S1**. Items endorsed in round one by scale and panel group (% agreement). **Table S2**. Items endorsed in round two by scale and panel group (% agreement). **Table S3**. Items endorsed in round three by scale and panel group (% agreement). **Table S4**. Items not endorsed in round one by scale and panel group (% agreement). **Table S5**. Items not endorsed in round two by scale and panel group (% agreement). **Table S6**. Items not endorsed in round three by scale and panel group (% agreement). (PDF 247 kb)

Additional file 2: T-tests comparing completers' and non-completers' responses on ten randomly selected items. **Table S1**. Comparison of study completers’ and non-completers’ round one responses on ten randomly selected items. (DOCX 16 kb)

Additional file 3: Completed guidelines for giving community presentations about eating disorders. (DOCX 72 kb)

## Abbreviations

ED/EDs: eating disorder/eating disorders
LE: lived experience
