# Supplementary material for: Development of guidelines for giving community presentations about eating disorders: a Delphi study
Source: J Eat Disord. 2017 Nov 21;5:54. doi: 10.1186/s40337-017-0183-x (PMC5697432; doi:10.1186/s40337-017-0183-x)
Supplement: Supplementary file 1 — Complete list of items endorsed or not endorsed by questionnaire round. Table S1. Items endorsed in round one by scale and panel group (% agreement). Table S2. Items endorsed in round two by scale and panel group (% agreement). Table S3. Items endorsed in round three by scale and panel group (% agreement). Table S4. Items not endorsed in round one by scale and panel group (% agreement). Table S5. Items not endorsed in round two by scale and panel group (% agreement). Table S6. Items not endorsed in round three by scale and panel group (% agreement). (PDF 247 kb) [file 40337_2017_183_MOESM1_ESM.pdf]

How should we talk about eating disorders?

A guide to giving eating disorder presentations in community settings.

Introduction

Purpose of these guidelines

These guidelines were developed for people who wish to give a presentation about eating disorders to community members (e.g., in the workplace, at awareness events, at universities, and at schools). They are intended to guide presentations to educate an audience, reduce the stigma of eating disorders, and promote help-seeking. They have been developed using the consensus of eating disorder professionals and advocates with lived experience through a rigorous research process.

> **A note on prevention**
>
> These guidelines are not intended for use as a prevention program. These guidelines are also not for use with children under 12 years old – presentations to younger children should focus on prevention only and not mention eating disorders.
>
> A prevention program aims to reduce the risk factors for eating disorders (e.g. body dissatisfaction, unhealthy weight control behaviours). Talking about eating disorder symptoms and behaviours is ineffective and can increase risk.
>
> It should be noted that if your presentation's purpose is to prevent eating disorders, research currently suggests that reducing risk factors (e.g. body dissatisfaction and dieting) and increasing protective factors (e.g., media literacy) is the most effective method. This can be achieved by using an evidence based prevention program that has already been written. We have provided recommendations for several programs in the 'prevention programs' section of these guidelines. Not using an evidence-based prevention program in adolescents (12-17) may be harmful.

In addition to helping you give an effective presentation about eating disorders, these guidelines can assist you in giving a *safe* presentation. Many researchers, community educators, and people with lived experience of eating disorders have expressed concern that including details about eating disorder symptoms and behaviours could harm people in the following ways (O'Dea, 2000):

- Introducing young people to concerns about dieting/weight control that did not exist previously;
- Glamourising eating disorders (for instance, using case studies of celebrities);
- Promoting fear of food through negative messages (e.g. sugar and fat are bad for you);
- Unintentionally conveying the presenter's own body dissatisfaction to the audience.

By discussing eating disorders in a safe way, we can still educate people about them, reduce stigma, and promote help-seeking.

How to use these guidelines

If you are planning a presentation about eating disorders, please read these guidelines first to ensure that you do no harm. These guidelines will help you make decisions about the sort of presentation you will give, and what content will feature in the presentation. We have also provided a list of organisations and educational resources at the back of this document for further information about eating disorders.

> There are a few terms that we will refer to throughout the document:
>
> *Helpful* – something that may reduce stigma and increase knowledge about eating disorders.
>
> *Harmful* – something that may increase stigma, provide incorrect knowledge, or cause/worsen eating disorder symptoms in the audience.
>
> *Speaker with lived experience* – a speaker with lived experience of an eating disorder who chooses to share their story with the audience.
>
> *Presenter* – Any person who is giving a presentation about eating disorders.
>
> *Adolescent* – People 12-17 years old.
>
> *Adult* – People 18 and over.

Contents

Preparing your presentation **1**

Qualities of a presenter 1

Before you begin... 1

Format of a presentation 1

Speakers who share their lived experience of an eating disorder 3

A note on recovery 3

How to avoid doing harm 3

Content of your presentation **4**

Media and Images 4

Talking about eating disorders in a non-stigmatising way 4

Talking about food and weight in a non-stigmatising way 4

Talking about symptoms 5

- How to discuss physical symptoms 5

- How to discuss behavioural symptoms 5

- How to discuss psychological symptoms 6

Risk factors and causes 7

Discussion that arises from your presentation **8**

Talking about recovery and help-seeking 8

How and where to seek help 8

List of resources for help-seeking information (eating disorder specific) **9**

List of resources for help-seeking information (general mental health) **10**

List of prevention resources **11**

Acknowledgements and how to cite these guidelines **12**

Preparing your presentation

Qualities of a presenter

Before planning a presentation, it is important to be aware of your own attitudes about weight/shape, food, exercise, and size. This is essential to ensure that you do not communicate any negative attitudes (e.g., encouraging dieting, size discrimination) to the audience. For instance, a presenter who suggests that you can't be healthy at a higher weight may encourage stigmatizing attitudes. To assist in communicating your message effectively, it is important that you are a good role model and advocate for a safe and respectful environment free from discrimination.

It may be helpful to invite a local professional trained in body image, self-esteem, eating disorders or mental health to assist with your presentation. A professional may be able to answer questions or provide extra information to both yourself and your audience.

There are several additional qualities that speakers with lived experience should be aware of when presenting their story. These are outlined in detail in the 'Lived experience speakers' section.

Before you begin…

As we outlined in the introduction, some content may trigger eating disorder behaviours in vulnerable people. Therefore, it is important to review presentations and materials for harmful or confusing information before the presentation is given. If you are planning to give the presentation on a regular basis, it is important to do this before each presentation and ensure your information is up to date.

You may wish to advise the audience that if the presentation raises any issues for them, information about appropriate sources of help will be provided at the end of the session.

Your presentation should include ideas and language that are age-appropriate. These guidelines will refer specifically to information that is appropriate for adolescent audiences and adult audiences.

Format of a presentation

Your presentation should have a clear purpose. The aims of your presentation will guide what information it needs to contain. For instance, a presentation that aims to educate the audience about eating disorders (improve mental health literacy) may contain factual information about the disorder and its causes and risk factors. A presentation aimed at reducing stigma might feature a speaker with lived experience.

The aims may also affect the number of sessions the presentation will run for. Single session presentations are common, but follow-up sessions can assist participants to implement strategies discussed.

When designing an engaging presentation, consider using a variety of ways to present the information to the audience, such as guest speakers, written material, audiovisual material, group discussions, or carefully selected resources from the internet.

Additional considerations (depending on the audience)

You should consider the gender of your audience and with a mixed audience it is likely to be helpful to design the content of a presentation to be gender neutral. (e.g., include a gender balance of case studies, warning signs, and prevalence statistics).

Adolescents

When presenting to adolescents, professionals suggest information should be interactive and participatory (e.g., group discussion, group activities and peer based learning).

Be aware that adolescents may feel uncomfortable discussing body image/eating disorders in front of the opposite gender. You may like to include small group activities that are separated by gender, and opportunities to ask questions anonymously after the presentation.

Adolescents can be particularly concerned about their body image and self-esteem and these are serious risk factors for the development of eating disorders. It may be helpful to provide information about body image and self-esteem in addition to eating disorders or within the presentation on eating disorders

Because adolescents are at increased risk of developing eating disorders, it is not recommended that speakers share their stories of lived experience.

Adults

While speakers sharing lived experience are not suitable for adolescents, you may wish to consider including an appropriate speaker with lived

experience for an adult audience. Speakers with lived experience can provide valuable information that may reduce eating disorder stigma, and provide a message that recovery is possible. It is essential for the safety of the speaker with lived experience that you do not push them to discuss or expand on areas/ topics they don't want to share, as this is likely to be harmful.

If you plan to have a speaker with lived experience, it is best that they are well supported by a mental health organisation with experience in providing community education programs about eating disorders. Additionally, you may like to invite a professional to be available for questions that may arise during the discussion period. For more information, see the section below.

Because body dissatisfaction and low self-esteem are important risk factors for eating disorders, you should consider incorporating information about body image and self-esteem in addition to eating disorders.

Speakers who share their lived experience of an eating disorder

If you choose to share your story of an eating disorder, you need to ensure that you take care of your own mental health and engage in self-care. A community mental health or eating disorder organisation with experience in providing community education programs about eating disorders can provide valuable support for speakers who decide to share their story. Please see page 9 of these guidelines for advice on who to contact about sharing your story. Before presenting, you should ensure that:

- You are committed to recovery before becoming an advocate for eating disorders awareness. This is helpful for your own self-care, as well as the safety of the audience;
- You consider the effects of self-disclosure on your personal well-being;
- You are provided with guidance by your supporting organisation before any speaking engagements;
- You have the opportunity to be debriefed by your supporting organisation after any speaking engagement;
- You decide what you are and are not willing to share before the presentation;
- You are aware that telling your story may bring up difficult emotions. If this happens, don't hesitate to ask for support from your supporting organisation or from a mental health professional;
- You are prepared to be asked unexpected questions.

How to avoid doing harm

It is a good idea for you to consider what you hope to achieve by sharing your story and ensure your presentation will do no harm.

Some ways to avoid doing harm:

- Carefully consider the potential impact of your message on the audience;
- Be aware of your own personal issues and attitudes around eating, weight and exercise so that you do not convey messages that could be harmful to the audience;
- There are several details that may be harmful to include when sharing your story. These are described under the headings of 'Causes and risk factors' (p.6), 'Physical symptoms' (p.5), 'Psychological symptoms'(p.5), 'Behavioural symptoms' (p.5), and 'Media and images' (p.4).

Helpful details and messages to include in a story of lived experience

It is important that you leave your audience with the message that there is hope, that recovery is possible, and that professional help is available. The following topics are likely to be helpful to include in your presentation:

- How you sought treatment – although keep in mind that you should not suggest that one particular treatment works or does not work for everyone.
- The methods that helped you to recover (e.g., therapy approach)
- The events and people that helped move you towards recovery.
- Interests, work, or relationships that have taken the place of your eating disorder
- What it feels like to be recovered.

You may be invited to answer questions from the audience. You are not expected to be an expert on all aspects of eating disorders, and should feel comfortable saying ‘I don’t know’ to any questions you don’t know the answers to. You may want to prepare to address common myths about eating disorders. **Some of the common myths are explained at http://www.nedc.com.au/myths, which include the factually correct information**. Not all people in the audience will have the same experiences or level of understanding as you, so it’s important for you to have respect for the experiences of others.

A note on recovery

Recovery from an eating disorder might not mean the same thing for everyone. Some people might feel recovered once their eating behaviour is normal again, whereas some might not feel recovered until they no longer experience negative thoughts about themselves. As the definition of ‘recovered’ can vary depending on the individual, it may be helpful to consult with a community educator who can help figure out whether sharing your story would be helpful at this stage in your recovery. It is helpful for you to be recovered for over a year before you share your story, both for yourself and the audience. A longer time in recovery also gives you more time to reflect upon your experiences, and will help you to educate the audience about the recovery process.

You should seek support from a mental health organisation (with experience in providing community education programs about eating disorders - see page 9) before sharing your story.

Content of your presentation

Media and images

Some types of media and images should NOT be included in presentations, for instance:

- Images of people before and after recovery from an eating disorder;
- Images of people with extreme weights or shapes (i.e., images of emaciated people);
- Media that identifies or names specific pro-eating disorder websites.

Before deciding on whether to include a documentary, ensure it doesn't contain harmful content as outlined in these guidelines. If you do choose to screen an appropriate documentary, you should include a Q&A session with mental health professionals. For adolescents, professionals recommend showing videos that promote body image media literacy (e.g., 'Miss Representation', Dove's 'Evolution') rather than specific stories about eating disorders.

Talking about eating disorders in a non-stigmatising way

The language used when talking about eating disorders can affect how the audience perceives people with eating disorders.

Messages and language that are likely to be helpful:

- Emphasising that no one is to blame for the development of an eating disorder;
- Explaining that eating disorders are serious mental illnesses, not choices;
- Explaining that eating disorders are serious without portraying them as hopeless;
- Describing the different types of eating disorders that exist;
- Using accurate terms for eating disorders (e.g., Anorexia Nervosa rather than 'extreme dieting');
- Explaining that all types of eating disorders are harmful to the individual, not just Anorexia Nervosa;
- Being aware of common myths about eating disorders (e.g., you have to be thin to have an eating disorder) and providing factually correct information (you can't tell by looking at someone if they have an eating disorder);
- Using messages and language that are respectful of people with eating disorders;
- Using language that does not define a person by their illness (e.g., use 'person with Bulimia') rather than language that does (e.g., 'that person is Bulimic');
- Including a message of hope that eating disorders can be overcome with the right treatment and support.

Messages and language that are likely to be harmful:

- Making eating disorders sound attractive or emphasising positive characteristics that may be associated with an eating disorder (e.g., will-power or self-control);
- Implying that all experiences of eating disorders are the same;
- Implying that all types of eating disorders are the same;
- Using judgemental or value-laden language (e.g., they are attention-seeking);
- Using language that trivialises eating disorders, (e.g., adding the suffix "orexia" to indicate an eating disorder, such as "bigorexia").

General tips for presentation language

Use positive language, such as "here are some options" rather than "you should do this". Using gender neutral language is also a good idea – for instance, ensuring that you do not only refer to people with eating disorders as female.

Special considerations for adult audiences

Professionals agreed that presentations advocating for increased treatment services and research in eating disorders may be helpful. This may depend on the aim of your presentation.

Talking about food and weight in a non-stigmatising way

Not all presentations about eating disorders need to educate the audience about food and weight. The way they are talked about, however, can have an impact on the audience.

Messages and language that are likely to be helpful include:

- Using language that does not focus on size or appearance specifically;
- Discouraging the idea that a particular body size leads to happiness;

- Actively challenging societal, peer, media and cultural pressures to lose weight;
- Explaining all foods can be eaten and enjoyed in moderation and avoid moral language to describe food (e.g. 'good' food, 'bad/junk' food);
- Explaining the harmful effects of fad diets.

Special considerations for athletes:

It may be useful to explain the relationship between good nutrition and performance when the presentation is to a group of athletes or dancers.

Talking about symptoms

Presentations should contain warning signs of eating disorders, as this may help audience members realise that they, or a loved one, have a problem. Page 7 contains a list of warning signs of eating disorders that were developed through an expert consensus study in 2008, and therefore are based on research evidence (please note: these may vary between individuals or across different types of eating disorders).

How to discuss physical symptoms

Ways of discussing physical symptoms that are likely to be helpful:

- Explaining that many people with eating disorders may be of average or above average weight and shape;
- Educating the audience about the physical consequences of an eating disorder (e.g. medical complications such as bone density loss or cardiac problems);
- Educating the audience about the possible dental consequences of eating disorders;
- Explaining the negative physical sensations that may occur when a person has an eating disorder, like fatigue and loss of co-ordination.

Ways of discussing physical symptoms that are likely to be harmful:

- Including specific body weights (i.e. lbs, kgs, BMI) of people with eating disorders;
- Including the amount of weight lost or gained by a person with an eating disorder;
- Mentioning specific clothing sizes of a person with an eating disorder.

Rather than discussing physical symptoms as primarily appearance-based, which can fuel the myth that you can tell someone has an eating disorder by looking at them, discussing physical symptoms as distinct from weight and appearance may help educate and reduce stigma.

How to discuss behavioural symptoms

Discussing behavioural symptoms of eating disorders can be difficult, in part due to concerns that they will normalise eating disorder behaviours or introduce vulnerable audience members to new methods of weight control.

Ways of discussing behaviours that are likely to be helpful:

- Explaining the dangers of weight control behaviours (e.g. laxatives, vomiting);
- Discussing the dangers of binge eating behaviours.

Ways of discussing behaviours that are likely to be harmful:

- Including 'numbers' when describing behaviours. For instance, the number of calories/kilojoules eaten by a person with an eating disorder, specific amounts of laxatives, or frequency or intensity of exercise done by a person with an eating disorder;
- Teaching or promoting calorie or kilojoule counting;
- Mentioning specific methods used in eating disorder behaviour (e.g. using fingers to purge);
- For adolescents (12-17), mentioning specific steps taken to disguise eating disorder behaviour (e.g. locking door to hide excessive exercise);
- For adolescents (12-17), mentioning the frequency, or any quantifiable details of an eating disorder.

How to discuss psychological symptoms

Ways of discussing psychological symptoms that are likely to be helpful:

- Including information about the psychological impact of an eating disorder (e.g., thoughts being taken over by weight and food, depression, fear/anxiety), and
- Discussing the social consequences of an eating disorder (e.g., disrupted friendships, isolation).

Special considerations for athletes:
When presenting to athletes, presenters may also wish to include information about how eating disorders will affect athletes specifically in order to encourage help-seeking:

- Explaining the negative consequences of eating disorders on performance, such as loss of muscular strength and endurance, decreased speed, loss of coordination, and poor judgement;
- Addressing ways in which extreme weight control behaviours (e.g. self-induced vomiting, laxative use) can hurt performance.

A note on self-harm behaviours

Some people with eating disorders engage in self-harm behaviours, and this may be tied to their behaviours and thoughts around food and weight. Avoid discussing specific details of self-harm (e.g. method or treatment) as this could be harmful for the audience. Discussing self-harm in general terms, if relevant to the presentation (e.g. 'I was very unwell and was engaging in self-harm') is likely to be more appropriate.

Risk factors and causes

When educating an audience about the causes of eating disorders, it is important not to oversimplify them. There are a range of risk factors for developing eating disorders, and the best way to explain this to an audience is to teach the biopsychosocial model. The biopsychosocial model states that a combination of biological (e.g., genetic), psychological (e.g., low self-esteem) and sociocultural factors (e.g., appearance-related teasing) contribute to the development of eating disorders. **You can find an explanation of major risk factors and protective factors at http://www.nedc.com.au/risk-factors.**

To help dispel myths around what causes an eating disorder, it is helpful to:

- Explain that eating disorders affect people regardless of their gender, race, ethnicity, socioeconomic status, or sexual orientation;
- Be careful not to portray eating disorders as caused by bad parenting;
- Be careful not to suggest that eating disorders develop due to celebrity culture and media. While some sociocultural factors can increase the risk for eating disorders (for instance, thin-ideal internalisation and body dissatisfaction), they cannot be attributed solely to the media.

Behavioural warning signs

- Dieting behaviours (e.g. fasting, counting calories/kilojoules, avoidance of food groups or types)
- Evidence of binge eating (e.g. disappearance or hoarding of food)
- Evidence of vomiting or laxative use (e.g. taking trips to the bathroom during or immediately after meals)
- Excessive, obsessive or ritualistic exercise patterns (e.g. exercising when injured or in bad weather, feeling compelled to perform a certain number of repetitions of exercises or experiencing distress if unable to exercise)
- Changes in food preferences (e.g. refusing to eat certain 'fatty' or 'bad' foods, cutting out whole food groups such as meat or dairy, claiming to dislike foods previously enjoyed, a sudden concern with 'healthy eating', or replacing meals with fluids)
- Development of rigid patterns around food selection, preparation and eating (e.g. cutting food into small pieces or eating very slowly)
- Avoidance of eating meals, especially when in a social setting (e.g. skipping meals by claiming they have already eaten or have an intolerance/allergy to particular foods)
- Lying about amount or type of food consumed or evading questions about eating and weight
- Behaviours focused on food (e.g. planning, buying, preparing and cooking meals for others but not actually consuming; interest in cookbooks, recipes and nutrition)
- Behaviours focused on body shape and weight (e.g. interest in weight-loss websites books and magazines, or images of thin people)
- Development of repetitive or obsessive behaviours relating to body shape and weight (e.g. body-checking such as pinching waist or wrists, repeated weighing of self, excessive time spent looking in mirrors)
- Social withdrawal or avoidance of previously enjoyed activities

Physical warning signs

- Weight loss or weight fluctuations
- Sensitivity to the cold or feeling cold most of the time, even in warm temperatures
- Changes in or loss of menstrual patterns
- Swelling around the cheeks or jaw, calluses on knuckles, or damage to teeth from vomiting
- Fainting

Psychological warning signs

- Pre-occupation with food, body shape and weight
- Extreme body dissatisfaction
- Distorted body image (e.g. complaining of being/feeling/looking fat when a healthy weight or underweight)
- Sensitivity to comments or criticism about exercise, food, body shape or weight
- Heightened anxiety around meal times
- Depression, anxiety or irritability
- Low self-esteem (e.g. negative opinions of self, feelings of shame, guilt or self-loathing)
- Rigid 'black and white' thinking (e.g. labelling of food as either 'good' or 'bad')

From Mental Health First Aid Australia Eating disorders: first aid guidelines. Melbourne: Mental Health First Aid Australia; 2008. [available from https://mhfa.com.au/sites/default/files/MHFA_eatdis_guidelines_A4_2013.pdf]

Discussion that arises from your presentation

Talking about recovery and help-seeking

To emphasise both the importance of help-seeking, and that recovery is possible, it can be helpful to include discussions about recovery. It is also essential to highlight help-seeking pathways for audience members who wish to help themselves or a loved one.

Each recovery story is different. Some people are able to recover without structured supports and treatment. However, it is important for the audience to know that most people who recover from an eating disorder do so with the support of both trained professionals and family/friends, rather than on their own. Different people can also benefit from different types of treatment, and there is not yet one type of treatment that is effective for all people with eating disorders. It's important for the audience to know that it is courageous and necessary to ask for help and support during recovery from an eating disorder.

How and where to seek help

Presentations should always include information about how to get help for an eating disorder, and should normalise help-seeking.

Before you give a presentation about eating disorders, ensure you have a written protocol for supporting and providing information to people who self-disclose during or after a presentation. Make sure you are prepared with information about eating disorder support services, and information on how to contact professionals specialising in eating disorders. Include information about general mental health support services, not only for those who self-disclose but for the entire audience. Explain that seeking help early can result in better recovery outcomes. However, it is also never too late to start getting help for an eating disorder.

We have included a list of resources in the back of this document that can give you information about seeking help (both for mental health and eating disorders, specifically) in your area.

Special consideration for adult audiences:

Depending on the aim of the presentation (e.g. to improve eating disorders mental health literacy), presenters may want to address how to recognise and prevent relapse.

Note for presentations given in high schools or universities/colleges

If a presentation is given in a school, you should provide opportunities for staff to talk if they have concerns and/or fears about how to support a student with an eating disorder. You may wish to hold a staff meeting and invite a mental health/eating disorder professional to assist you.

Help-seeking and further information: List of major eating disorder organisations

The following organisations can provide you with information on eating disorders, as well as how to get help for an eating disorder.

Australia

National Eating Disorders Collaboration

Website: www.nedc.com.au

The Butterfly Foundation

Website: www.thebutterflyfoundation.org.au

Canada

National Eating Disorders Information Centre

Website: www.nedic.ca

Ireland

Bodywhys

Website: www.bodywhys.ie

New Zealand

EDANZ

Website: www.ed.org.nz/

United Kingdom

Beat UK

Website: www.b-eat.co.uk

United States

National Eating Disorders Association

Website: www.nationaleatingdisorders.org/find-help-support

Note for people with lived experience who wish to share their story: The Butterfly Foundation, National Eating Disorders Information Centre, Bodywhys, Beat UK, and National Eating Disorders Association can provide you with information and advice on sharing your story with an audience.

Help-seeking and further information: List of major mental health organisations

Australia
Beyond Blue
Website: www.beyondblue.org.au

Headspace (for people 12-25)
Website: headspace.org.au/

Canada
Canadian Mental Health Association
Website: www.cmha.ca/

Mind Your Mind (for adolescents and young people)
Website: mindyourmind.ca/

Ireland
Mental Health Ireland
Website: www.mentalhealthireland.ie/

Jigsaw (For young people)
Website: www.jigsaw.ie/

New Zealand
Mental Health Foundation of New Zealand
Website: www.mentalhealth.org.nz/

United Kingdom
Mind
Website: www.mind.org.uk/

YoungMinds (For children and young people)
Website: www.youngminds.org.uk/

United States
Mental Health America
Website: www.mentalhealthamerica.net/

ReachOut US (for teens and young people)
Website: us.reachout.com/

Prevention resources

The following resources are evidence-based, researcher developed, prevention programs.

For young children:

Confident Body, Confident Child

To help parents create a family environment in which children aged 2-6 years can develop healthy body image and eating patterns.

Website: http://www.latrobe.edu.au/psychology/research/research-areas/clinical-and-health-psychology/confident-body-study

Reference: Hart, L. M., Damiano, S. R. and Paxton, S. J. (2016), Confident body, confident child: A randomized controlled trial evaluation of a parenting resource for promoting healthy body image and eating patterns in 2- to 6-year old children. *International Journal of Eating Disorders, 49*: 458–472. doi:10.1002/eat.22494

For adolescents:

Happy Being Me

Co-educational school-based body image intervention for early high school. For program workbooks contact Professor Susan Paxton (susan.paxton@latrobe.edu.au)

Reference: Dunstan, C.J., Paxton, S.J., & McLean, S.A. (in press). An evaluation of a body image intervention in adolescent girls delivered in single-sex versus co-educational classroom settings. *Eating Behaviors.* http://dx.doi.org/10.1016/j.eatbeh.2016.03.016

Dove Self-Esteem Project

Materials for teachers and parents
Website: http://selfesteem.dove.co.uk

Research:

MediaSmart

Co-educational school-based body image intervention for early high school

Website: http://www.flinders.edu.au/sabs/psychology/services/flinders-university-services-for-eating-disorders/mediasmart/

Research: Wilksch, S. M., Tiggemann, M. and Wade, T. D. (2006), Impact of interactive school-based media literacy lessons for reducing internalization of media ideals in young adolescent girls and boys. *International Journal of Eating Disorders, 39*, 385–393. doi:10.1002/eat.20237

For late adolescents and young adults:

The Body Project

Website: http://www.bodyprojectsupport.org/resources/materials

Research: Stice., E., Butryn, M.L., Rohde, P & Shaw, H. (2013). An effectiveness trial of a new enhanced dissonance eating disorder prevention program among female college students. *Behaviour Research and Therapy*, *51,* 862-871
