# Supplementary material for: Development of guidelines for giving community presentations about eating disorders: a Delphi study
Source: J Eat Disord. 2017 Nov 21;5:54. doi: 10.1186/s40337-017-0183-x (PMC5697432; doi:10.1186/s40337-017-0183-x)
Supplement: Supplementary file 2 — T-tests comparing completers' and non-completers' responses on ten randomly selected items. Table S1. Comparison of study completers’ and non-completers’ round one responses on ten randomly selected items. (DOCX 16 kb) [file 40337_2017_183_MOESM2_ESM.docx]

**Supplementary Material 3**

Table 1. *Comparison of study completers’ and non-completers’ round one responses on ten randomly selected items*

| Item | Completers  M (SD) | Non-completers  M (SD) | n (completers/non-completers) | *t* | df | *p* |
| --- | --- | --- | --- | --- | --- | --- |
| Authors SHOULD… invite a professional to be available for questions that may arise during the discussion period, if someone is presenting a lived experience story ^a, c^ | 1.92 (.86) | 2.00 (.60) | 13/12 | -.256 | 23 | ns |
| Authors and presenters SHOULD... emphasise that no one is to blame for the development of an eating disorder ^b, d^ | 1.42 (.90) | 1.17 (.39) | 12/12 | .883 | 22 | ns |
| Authors and presenters SHOULD... explain the dangers of weight control behaviours (i.e. laxatives, vomiting) ^a, c^ | 1.92 (1.12) | 1.75 (.87) | 13/12 | .431 | 23 | ns |
| LE Speakers SHOULD NOT... specify the frequency or duration  they engaged in eating disorder behaviours ^a, d^ | 2.00 (1.22) | 2.85 (1.82) | 13/13 | -1.391 | 21.03 | ns |
| Authors and presenters SHOULD NOT... discuss the symptoms and detrimental effects of eating disorders when the intention of the presentation is prevention, as there is not sufficient evidence to suggest that this is effective. ^b, d^ | 3.00 (.60) | 2.92 (1.38) | 12/13 | .183 | 16.69 | ns |
| Presentations SHOULD NOT… teach or promote calorie/kilojoule counting ^b, d^ | 3.00 (1.86) | 2.83 (1.80) | 12/12 | .223 | 22 | ns |
| Presentations SHOULD NOT include images of people before and after recovery from an eating disorder ^a, c^ | 1.62 (1.04) | 1.67 (1.37) | 13/12 | -.106 | 23 | ns |
| Authors SHOULD... review presentations and materials for ambiguity and risk of harm on a regular basis ^a, d^ | 1.54 (.52) | 1.08 (.28) | 13/13 | 2.828 | 18.34 | .01* |
| Authors SHOULD... be aware that females and males may feel uncomfortable discussing body image/eating disorders in front of the opposite gender ^a, d^ | 2.31 (.95) | 1.77 (.73) | 13/13 | 1.627 | 24 | ns |
| Authors SHOULD... consider using an appropriate speaker with lived experience ^b, c^ | 1.92 (1.00) | 1.67 (.98) | 12/12 | .618 | 22 | ns |

*statistically significant difference
^a^ This item was rated for adolescent audiences

^b^ This item was rated for adult audiences

^c^ This item was rated for its inclusion in the guidelines

^d^ This item was rated for its helpfulness/potential to do harm
