# Supplementary material for: Development of guidelines for giving community presentations about eating disorders: a Delphi study
Source: J Eat Disord. 2017 Nov 21;5:54. doi: 10.1186/s40337-017-0183-x (PMC5697432; doi:10.1186/s40337-017-0183-x)
Supplement: Supplementary file 3 — Completed guidelines for giving community presentations about eating disorders. (DOCX 72 kb) [file 40337_2017_183_MOESM3_ESM.docx]

**Supplementary Material 1**

Table 1*. Items endorsed in round one by scale and panel group (% agreement)*

|  | *Should this be included in guidelines?* | | *How helpful/harmful would this be?* | |
| --- | --- | --- | --- | --- |
| *Statement* | Professionals | Advocates | Professionals | Advocates |
| Authors SHOULD… provide information about body image and self-esteem within their presentation on eating disorders (12-17) | 90.91 | 85.71 | 100.00 | 85.71 |
| Authors SHOULD… consider inviting a local professional trained in body image/self-esteem/eating disorders or mental health (18+) | 81.82 | 86.67 | 90.00 | 86.67 |
| When designing the presentation, authors SHOULD... consider multiple ways to disseminate information to the audience, such as seminars, guest speakers, written material, audiovisual, or use of the internet (18+) | 100.00 | 93.33 | 100.00 | 80.00 |
| Authors and presenters SHOULD... be aware of their own attitudes about weight/shape, food, exercise,and size and ensure that they do not communicate any negative attitudes (e.g. body dissatisfaction, size discrimination) to their audience (12-17) | 100.00 | 100.00 | 88.89 | 84.62 |
| Authors and presenters SHOULD... be aware of their own attitudes about weight/shape, food, exercise,and size and ensure that they do not communicate any negative attitudes (e.g. body dissatisfaction, size discrimination) to their audience (18+) | 90.91 | 100.00 | 88.89 | 85.71 |
| Authors and presenters SHOULD... be good role models and advocate for a safe and respectful environment free from discrimination (12-17) | 100.00 | 100.00 | 100.00 | 100.00 |
| Authors and presenters SHOULD... be good role models and advocate for a safe and respectful environment free from discrimination (18+) | 100.00 | 100.00 | 100.00 | 100.00 |
| LE Speakers SHOULD… be provided guidance by their supporting organisation before any speaking engagements (12-17)^a^ | 100.00 | 100.00 | 100.00 | 100.00 |
| LE Speakers SHOULD… be provided guidance by their supporting organisation before any speaking engagements (18+) | 100.00 | 93.33 | 100.00 | 93.33 |
| LE Speakers SHOULD... have the opportunity to be debriefed after any speaking engagement (12-17) ^a^ | 100.00 | 100.00 | 90.00 | 100.00 |
| LE Speakers SHOULD... have the opportunity to be debriefed after any speaking engagement (18+) | 100.00 | 100.00 | 90.00 | 100.00 |
| LE Speakers SHOULD... consider the effects of self-disclosure on their personal well-being (12-17) ^a^ | 100.00 | 100.00 | 100.00 | 92.86 |
| LE Speakers SHOULD... consider the effects of self-disclosure on their personal well-being (18+) | 100.00 | 100.00 | 100.00 | 93.33 |
| LE Speakers SHOULD... be aware that talking about their experience may bring up difficult emotions for them (12-17) ^a^ | 100.00 | 100.00 | 100.00 | 85.71 |
| LE Speakers SHOULD... be aware that talking about their experience may bring up difficult emotions for them (18+) | 100.00 | 100.00 | 100.00 | 86.67 |
| LE Speakers SHOULD... decide what they are and what they are not willing to share before the presentation (12-17) ^a^ | 100.00 | 100.00 | 100.00 | 92.86 |
| LE Speakers SHOULD... decide what they are and what they are not willing to share before the presentation (18+) | 100.00 | 100.00 | 100.00 | 93.33 |
| LE Speakers SHOULD... be aware that they may be asked unexpected questions (12-17) ^a^ | 100.00 | 100.00 | 100.00 | 92.86 |
| LE Speakers SHOULD... be aware that they may be asked unexpected questions (18+) | 100.00 | 100.00 | 100.00 | 93.33 |
| LE Speakers SHOULD... have respect for the experiences of the people in the audience (12-17)^a^ | 100.00 | 100.00 | 100.00 | 92.86 |
| LE Speakers SHOULD... have respect for the experiences of the people in the audience (18+) | 100.00 | 100.00 | 100.00 | 93.33 |
| LE Speakers SHOULD  carefully consider the potential impact of their message on the audience (12-17) ^a^ | 81.82 | 92.86 | 90.00 | 92.31 |
| LE Speakers SHOULD  carefully consider the potential impact of their message on the audience (18+) | 81.82 | 93.33 | 90.00 | 92.86 |
| LE Speakers SHOULD NOT…answer questions that they do not know the answers to (12-17) ^a^ | 90.91 | 92.86 | 88.89 | 92.86 |
| LE Speakers SHOULD NOT…answer questions that they do not know the answers to (18+) | 90.91 | 93.33 | 90.00 | 93.33 |
| LE Speakers SHOULD NOT… suggest that a particular treatment works for everyone (12-17) ^a^ | 100.00 | 92.86 | 90.00 | 92.31 |
| LE Speakers SHOULD NOT… suggest that a particular treatment works for everyone (18+) | 100.00 | 86.67 | 90.00 | 86.67 |
| LE Speakers SHOULD... consider what they hope to achieve by sharing their story and ensure their presentation will do no harm (12-17) ^a^ | 100.00 | 100.00 | 100.00 | 100.00 |
| LE Speakers SHOULD... consider what they hope to achieve by sharing their story and ensure their presentation will do no harm (18+) | 100.00 | 100.00 | 100.00 | 93.33 |
| LE Speakers SHOULD… leave their audience with the message that there is hope, and recovery from food and weight concerns is possible (12-17) ^a^ | 100.00 | 84.62 | 100.00 | 92.31 |
| LE Speakers SHOULD… leave their audience with the message that there is hope, and recovery from food and weight concerns is possible (18+) | 100.00 | 85.71 | 100.00 | 92.86 |
| LE Speakers SHOULD… leave their audience with the message that professional help is available (12-17) ^a^ | 100.00 | 92.86 | 100.00 | 92.86 |
| LE Speakers SHOULD… leave their audience with the message that professional help is available (18+) | 100.00 | 93.33 | 100.00 | 93.33 |
| LE Speakers SHOULD... be aware of their own personal issues and attitudes around eating, weight and exercise so that they do not convey messages that could be harmful to the audience (12-17) ^a^ | 100.00 | 100.00 | 100.00 | 92.86 |
| LE Speakers SHOULD... be aware of their own personal issues and attitudes around eating, weight and exercise so that they do not convey messages that could be harmful to the audience (18+) | 100.00 | 93.33 | 100.00 | 93.33 |
| LE speakers SHOULD... discuss how they sought treatment (12-17) ^a^ | 100.00 | 85.71 | 100.00 | 92.86 |
| LE speakers SHOULD... discuss how they sought treatment (18+) | 100.00 | 86.67 | 100.00 | 93.33 |
| LE speakers SHOULD… discuss what methods helped them to recover (12-17) ^a^ | 81.82 | 85.71 | 90.00 | 85.71 |
| LE speakers SHOULD… discuss what methods helped them to recover (18+) | 81.82 | 85.71 | 90.00 | 86.67 |
| LE Speakers SHOULD… talk about what it felt like to be recovered (12-17) ^a^ | 100.00 | 92.86 | 100.00 | 92.86 |
| LE Speakers SHOULD… talk about what it felt like to be recovered (18+) | 100.00 | 92.86 | 100.00 | 93.33 |
| Authors and presenters SHOULD... use positive language, such as "here are some options" rather than "you should do this" (12-17) | 90.91 | 85.71 | 90.00 | 92.86 |
| Authors and presenters SHOULD... use positive language, such as "here are some options" rather than "you should do this" (18+) | 90.91 | 86.67 | 90.00 | 93.33 |
| Authors and presenters SHOULD... use gender neutral language (18+) | 90.91 | 86.67 | 90.00 | 80.00 |
| Authors and presenters SHOULD... emphasise that no one is to blame for the development of an eating disorder (12-17) | 100.00 | 100.00 | 90.00 | 100.00 |
| Authors and presenters SHOULD... emphasise that no one is to blame for the development of an eating disorder (18+) | 100.00 | 100.00 | 90.00 | 100.00 |
| Authors and presenters SHOULD… be respectful of people with eating disorders (12-17) | 100.00 | 92.86 | 100.00 | 92.86 |
| Authors and presenters SHOULD… be respectful of people with eating disorders (18+) | 100.00 | 93.33 | 100.00 | 93.33 |
| Authors SHOULD... review presentations and materials for ambiguity and risk of harm before the presentation is given. (12-17) | 100.00 | 100.00 | 90.00 | 92.31 |
| Authors SHOULD... review presentations and materials for ambiguity and risk of harm before the presentation is given. (18+) | 100.00 | 100.00 | 88.89 | 100.00 |
| Authors SHOULD... review presentations and materials for ambiguity and risk of harm on a regular basis (12-17) | 100.00 | 92.86 | 90.00 | 85.71 |
| Authors SHOULD... review presentations and materials for ambiguity and risk of harm on a regular basis (18+) | 100.00 | 100.00 | 90.00 | 93.33 |
| Presentations SHOULD... include ideas and language that are age-appropriate (12-17) | 100.00 | 92.86 | 100.00 | 85.71 |
| Presentations SHOULD... include ideas and language that are age-appropriate (18+) | 90.91 | 80.00 | 90.00 | 80.00 |
| Presentations SHOULD… contain the message that eating disorders are serious mental illnesses, not choices (12-17) | 100.00 | 100.00 | 100.00 | 92.86 |
| Presentations SHOULD… contain the message that eating disorders are serious mental illnesses, not choices (18+) | 100.00 | 100.00 | 100.00 | 93.33 |
| Presentations SHOULD… contain the message that eating disorders are serious without portraying them as hopeless (12-17) | 100.00 | 92.86 | 100.00 | 92.86 |
| Presentations SHOULD… contain the message that eating disorders are serious without portraying them as hopeless (18+) | 100.00 | 100.00 | 100.00 | 93.33 |
| Presentations SHOULD... contain a message of hope, that eating disorders can be overcome with the right treatment and support (12-17) | 100.00 | 85.71 | 100.00 | 85.71 |
| Presentations SHOULD... contain a message of hope, that eating disorders can be overcome with the right treatment and support (18+) | 100.00 | 86.67 | 100.00 | 86.67 |
| Authors, and presenters SHOULD... be aware of common myths about eating disorders and provide factually correct information (e.g. you have to be thin to have an eating disorder) (12-17) | 100.00 | 92.86 | 100.00 | 92.31 |
| Authors, and presenters SHOULD... be aware of common myths about eating disorders and provide factually correct information (e.g. you have to be thin to have an eating disorder) (18+) | 100.00 | 100.00 | 100.00 | 92.86 |
| Authors and presenters SHOULD…explain that all types of eating disorders are harmful to the individual, not just Anorexia Nervosa (18+) | 100.00 | 93.33 | 100.00 | 86.67 |
| Presentations SHOULD… describe the different types of eating disorders that exist (18+) | 90.91 | 86.67 | 90.00 | 86.67 |
| Authors and presenters SHOULD… explain that many people with eating disorders may be of average or above average weight and shape (18+) | 90.91 | 80.00 | 80.00 | 80.00 |
| Presentations SHOULD… include facts about the possible dental consequences of eating disorders (12-17) | 90.91 | 85.71 | 80.00 | 85.71 |
| Presentations SHOULD… include facts about the possible dental consequences of eating disorders (18+) | 90.91 | 80.00 | 80.00 | 86.67 |
| Presentations SHOULD… contain information about the negative physical sensations that may occur when a person has an eating disorder, like fatigue and loss of co-ordination (12-17) | 90.91 | 85.71 | 90.00 | 85.71 |
| Presentations SHOULD… contain information about the negative physical sensations that may occur when a person has an eating disorder, like fatigue and loss of co-ordination (18+) | 90.91 | 80.00 | 90.00 | 86.67 |
| Presentations SHOULD… include information about the psychological impact of an eating disorder, (e.g. thoughts being taken over by weight and food, depression, fear) (18+) | 100.00 | 93.33 | 80.00 | 86.67 |
| Authors and presenters SHOULD…explain the social consequences of an eating disorder (e.g. disrupted friendships, isolation) (12-17) | 90.91 | 85.71 | 100.00 | 85.71 |
| Authors and presenters SHOULD…explain the social consequences of an eating disorder (e.g. disrupted friendships, isolation) (18+) | 90.91 | 100.00 | 100.00 | 86.67 |
| Presentations SHOULD…emphasise the warning signs of eating disorders that friends or family may observe (12-17) | 100.00 | 85.71 | 90.00 | 92.86 |
| Presentations SHOULD…emphasise the warning signs of eating disorders that friends or family may observe (18+) | 90.91 | 80.00 | 90.00 | 86.67 |
| Presentations SHOULD... include warning signs of eating disorders (12-17) | 100.00 | 92.86 | 100.00 | 100.00 |
| Presentations SHOULD... include warning signs of eating disorders (18+) | 100.00 | 100.00 | 100.00 | 100.00 |
| Authors and presenters SHOULD... explain  that a combination of biological, psychological  and sociocultural factors contribute to the development of eating disorders (12-17) | 100.00 | 100.00 | 100.00 | 100.00 |
| Authors and presenters SHOULD... explain  that a combination of biological, psychological  and sociocultural factors contribute to the development of eating disorders (18+) | 100.00 | 100.00 | 100.00 | 100.00 |
| Authors and presenters SHOULD... explain that eating disorders affect people regardless of their gender, race, ethnicity, socioeconomic status, or sexual orientation (12-17) | 100.00 | 100.00 | 100.00 | 100.00 |
| Authors and presenters SHOULD... explain that eating disorders affect people regardless of their gender, race, ethnicity, socioeconomic status, or sexual orientation (18+) | 100.00 | 100.00 | 100.00 | 100.00 |
| Presentations to parents SHOULD…discuss the potential harm of appearance related teasing (12-17) | 100.00 | 92.86 | 100.00 | 92.86 |
| Presentations to parents should… explain the negative impact of appearance related teasing between siblings (12-17) | 100.00 | 92.86 | 100.00 | 100.00 |
| Authors and presenters SHOULD... explain that each recovery story is different (12-17) | 100.00 | 100.00 | 90.00 | 100.00 |
| Authors and presenters SHOULD... explain that each recovery story is different (18+) | 100.00 | 100.00 | 90.00 | 100.00 |
| Authors and presenters SHOULD… explain that there is not one type of treatment that is effective for all people with eating disorders (12-17) | 100.00 | 100.00 | 90.00 | 100.00 |
| Authors and presenters SHOULD… explain that there is not one type of treatment that is effective for all people with eating disorders (18+) | 100.00 | 100.00 | 90.00 | 100.00 |
| Authors and presenters SHOULD... tell the audience that it is  courageous and necessary to ask for help and support during recovery from an eating disorder (12-17) | 100.00 | 100.00 | 100.00 | 100.00 |
| Authors and presenters SHOULD... tell the audience that it is  courageous and necessary to ask for help and support during recovery from an eating disorder (18+) | 100.00 | 100.00 | 100.00 | 100.00 |
| Presentations SHOULD... include information about eating disorder support services (12-17) | 100.00 | 100.00 | 100.00 | 100.00 |
| Presentations SHOULD... include information about eating disorder support services (18+) | 100.00 | 100.00 | 100.00 | 100.00 |
| Presentations SHOULD... include information about general mental health support services (12-17) | 100.00 | 92.31 | 100.00 | 92.31 |
| Presentations SHOULD... include information about general mental health support services (18+) | 100.00 | 92.86 | 100.00 | 92.86 |
| Presentations SHOULD... include information on how to contact professionals specialising in eating disorders (12-17) | 100.00 | 100.00 | 100.00 | 92.31 |
| Presentations SHOULD... include information on how to contact professionals specialising in eating disorders (18+) | 100.00 | 100.00 | 100.00 | 92.86 |
| Authors SHOULD…have a written protocol for supporting and providing information to persons who self-disclose during or after a presentation (12-17) | 80.00 | 100.00 | 100.00 | 100.00 |
| Authors SHOULD…have a written protocol for supporting and providing information to persons who self-disclose during or after a presentation (18+) | 81.82 | 100.00 | 100.00 | 100.00 |
| Presentations SHOULD... normalise help-seeking (12-17) | 100.00 | 100.00 | 100.00 | 100.00 |
| Presentations SHOULD... normalise help-seeking (18+) | 100.00 | 100.00 | 100.00 | 100.00 |
| In a school presentation, presenters SHOULD... provide opportunities for staff to talk if they have concerns and/or fears about how to support a student with an eating disorder (12-17) | 81.82 | 100.00 | 90.00 | 100.00 |
| In a school presentation, presenters SHOULD... provide opportunities for staff to talk if they have concerns and/or fears about how to support a student with an eating disorder (18+) | 81.82 | 92.86 | 90.00 | 92.86 |
| Presentations SHOULD... explain the relationship between good nutrition and performance (12-17) | 81.82 | 92.31 | 90.00 | 84.62 |
| Presentations SHOULD... explain the relationship between good nutrition and performance (18+) | 81.82 | 85.71 | 90.00 | 85.71 |

^a^ Speakers with lived experience not endorsed for this age group.

Table 2*. Items endorsed in round two by scale and panel group (% agreement)*

|  | *Should this be included in guidelines?* | | *How helpful/harmful would this be?* | |
| --- | --- | --- | --- | --- |
| *Statement* | Professionals | Advocates | Professionals | Advocates |
| Authors SHOULD... consider using an appropriate speaker with lived experience (18+) | 85.71 | 80.00 | 80.00 | 93.33 |
| Authors SHOULD... be aware that if they plan to have a speaker with lived experience, it is best that the speaker be supported (e.g. given guidance for how to tell their story, provided with debriefing following their presentation) by a mental health organisation with experience in providing community education programs about eating disorders (12-17) ^a^ | 100.00 | 100.00 | 85.71 | 90.00 |
| Having a speaker with lived experience supported (e.g. given guidance for how to tell their story, provided with debriefing following their presentation) by a mental health organisation with experience in providing community education programs about eating disorders is likely to be... (for the speaker)^b^ |  |  | 100.00 | 85.71 |
| The author/presenter SHOULD NOT... push LE speakers to discuss or expand on areas/topics they don't want to share (12-17) | 90.91 | 92.86 | 85.71 (HARMFUL) | 100.00 (HARMFUL) |
| The author/presenter SHOULD NOT... push LE speakers to discuss or expand on areas/topics they don't want to share (18+) | 90.91 | 93.33 | 85.71 (HARMFUL) | 90.00 (HARMFUL) |
| Pushing speakers with lived experience to discuss or expand on areas/topics they don't want to share is likely to be... (for the speaker) |  |  | 100.00 (HARMFUL) | 100.00 (HARMFUL) |
| Authors and presenters SHOULD... have a clear purpose for designing and delivering their presentation, because the aims will guide what information it needs to contain (12-17) ^c^ | 100.00 | 100.00 |  |  |
| Authors and presenters SHOULD... have a clear purpose for designing and delivering their presentation, because the aims will guide what information it needs to contain (18+) ^c^ | 100.00 | 100.00 |  |  |
| Authors and presenters SHOULD... be aware that if their purpose is to PREVENT eating disorders, research currently suggests that reducing risk factors (e.g. body dissatisfaction and dieting) is the most effective method (12-17) ^c^ | 100.00 | 90.00 |  |  |
| Authors and presenters SHOULD... be aware that if their purpose is to PREVENT eating disorders, research currently suggests that reducing risk factors (e.g. body dissatisfaction and dieting) is the most effective method (18+) ^c^ | 100.00 | 90.00 |  |  |
| Speakers with lived experience SHOULD... be committed to their recovery before becoming an advocate for eating disorders awareness (12-17) | 100.00 | 90.00 | 100.00 | 100.00 |
| Speakers with lived experience SHOULD... be committed to their recovery before becoming an advocate for eating disorders awareness (18+) | 100.00 | 90.00 | 100.00 | 100.00 |
| Speakers being committed to their recovery before becoming an advocate for eating disorders awareness is likely to be... (for the speaker) |  |  | 100.00 | 100.00 |
| LE speakers SHOULD... be prepared to address common myths about eating disorders (12-17) | 85.71 | 90.00 | 85.71 | 100.00 |
| LE speakers SHOULD... be prepared to address common myths about eating disorders (18+) | 85.71 | 100.00 | 85.71 | 100.00 |
| LE speakers SHOULD NOT… discuss specific details of self-harm (e.g. method or treatment) (12-17) | 100.00 | 90.00 | 100.00 (HARMFUL) | 100.00 (HARMFUL) |
| LE speakers SHOULD NOT… discuss specific details of self-harm (e.g. method or treatment) (18+) | 100.00 | 90.00 | 100.00 (HARMFUL) | 90.00 (HARMFUL) |
| LE Speakers SHOULD... talk about the events that helped move them towards recovery (12-17) | 100.00 | 90.00 | 100.00 | 90.00 |
| LE Speakers SHOULD... talk about the events that helped move them towards recovery (18+) | 100.00 | 90.00 | 100.00 | 80.00 |
| LE Speakers SHOULD... talk about the people who helped move them towards recovery (18+) | 85.71 | 80.00 | 100.00 | 80.00 |
| LE speakers SHOULD... talk about interests, work, or relationships that have taken the place of their eating disorder (12-17) | 85.71 | 80.00 | 85.71 | 90.00 |
| LE speakers SHOULD... talk about interests, work, or relationships that have taken the place of their eating disorder (18+) | 85.71 | 90.00 | 85.71 | 100.00 |
| Presentations SHOULD NOT... include images of people before and after recovery from an eating disorder (12-17) | 100.00 | 90.00 | 85.71 (HARMFUL) | 80.00 (HARMFUL) |
| Presentations SHOULD NOT… name any specific pro-eating disorder websites (12-17) | 90.91 | 92.86 | 100.00 (HARMFUL) | 90.00 (HARMFUL) |
| Presentations SHOULD NOT… name any specific pro-eating disorder websites (18+) | 81.82 | 86.67 | 100.00 (HARMFUL) | 90.00 (HARMFUL) |
| Authors and presenters SHOULD... use gender neutral language (12-17) | 81.82 | 85.71 | 100.00 | 100.00 |
| Presentations SHOULD NOT…make eating disorders sound attractive or emphasise positive characteristics that may be associated with an eating disorder e.g. will-power or self-control (12-17) | 90.91 | 85.71 | 100.00 (HARMFUL) | 100.00 (HARMFUL) |
| Presentations SHOULD NOT…make eating disorders sound attractive or emphasise positive characteristics that may be associated with an eating disorder e.g. will-power or self-control (18+) | 90.91 | 86.67 | 100.00 (HARMFUL) | 90.00 (HARMFUL) |
| Authors and presenters SHOULD NOT... imply that all types of eating disorders are the same (18+) | 90.91 | 80.00 | 85.71 (HARMFUL) | 100.00 (HARMFUL) |
| Authors and presenters SHOULD NOT... imply that all experiences of eating disorders are the same (12-17) | 100.00 | 100.00 | 100.00 | 90.00 (HARMFUL) |
| Authors and presenters SHOULD NOT... imply that all experiences of eating disorders are the same (18+) | 100.00 | 100.00 | 100.00 | 90.00 (HARMFUL) |
| Authors and presenters SHOULD ... consider using language that does not focus on size or appearance specifically (12-17) | 100.00 | 90.00 | 100.00 | 90.00 |
| Authors and presenters SHOULD ... consider using language that does not focus on size or appearance specifically (18+) | 100.00 | 90.00 | 100.00 | 90.00 |
| Authors and presenters SHOULD... be aware that some content may trigger eating disorder behaviour (12-17) | 100.00 | 92.86 | 100.00 | 100.00 |
| Authors and presenters SHOULD... be aware that some content may trigger eating disorder behaviour (18+) | 100.00 | 93.33 | 100.00 | 100.00 |
| Presentations SHOULD… include a warning that content may be triggering for vulnerable people (i.e. trigger warning) (12-17) | 85.71 | 100.00 | 85.71 | 90.00 |
| Presentations SHOULD… include a warning that content may be triggering for vulnerable people (i.e. trigger warning) (18+) | 85.71 | 90.00 | 85.71 | 90.00 |
| Authors and presenters SHOULD… discourage the idea that a particular body size leads to happiness (12-17) | 90.91 | 92.86 | 85.71 | 80.00 |
| Authors and presenters SHOULD… discourage the idea that a particular body size leads to happiness (18+) | 90.91 | 93.33 | 85.71 | 90.00 |
| Authors and presenters SHOULD… actively challenge societal, peer, media and cultural pressures to lose weight (12-17) | 100.00 | 80.00 | 100.00 | 90.00 |
| Authors and presenters SHOULD… actively challenge societal, peer, media and cultural pressures to lose weight (18+) | 100.00 | 80.00 | 100.00 | 90.00 |
| Authors and presenters SHOULD explain that all foods can be eaten and enjoyed in moderation (12-17) | 100.00 | 90.00 | 85.71 | 90.00 |
| Authors and presenters SHOULD explain that all foods can be eaten and enjoyed in moderation (18+) | 81.82 | 80.00 | 85.71 | 90.00 |
| Presentations SHOULD NOT... contain judgemental or value-laden language (e.g. they are attention-seeking) (12-17) | 100.00 | 85.71 | 100.00 (HARMFUL) | 100.00 (HARMFUL) |
| Presentations SHOULD NOT... contain judgemental or value-laden language (e.g. they are attention-seeking) (18+) | 100.00 | 86.67 | 100.00 (HARMFUL) | 100.00 (HARMFUL) |
| Presentations SHOULD NOT ... use moral language to describe food (e.g. 'good' food, 'bad/junk' food) (12-17) | 85.71 | 90.00 | 85.71 (HARMFUL) | 90.00 (HARMFUL) |
| Presentations SHOULD NOT ... use moral language to describe food (e.g. 'good' food, 'bad/junk' food) (18+) | 100.00 | 85.71 | 85.71 (HARMFUL) | 90.00 (HARMFUL) |
| Authors and presenters SHOULD…explain that all types of eating disorders are harmful to the individual, not just Anorexia Nervosa (12-17) | 100.00 | 100.00 | 100.00 | 100.00 |
| Presentations SHOULD NOT… include specific body weights (i.e. lbs, kgs, BMI) of people with eating disorders (12-17) | 85.71 | 90.00 | 100.00 (HARMFUL) | 90.00 (HARMFUL) |
| Presentations SHOULD NOT… include specific body weights (i.e. lbs, kgs, BMI) of people with eating disorders (18+) | 85.71 | 90.00 | 100.00 (HARMFUL) | 90.00 (HARMFUL) |
| Presentations SHOULD NOT… include amount of weight lost by a person with an eating disorder (18+) | 81.82 | 80.00 | 100.00 (HARMFUL) | 80.00 (HARMFUL) |
| Presentations SHOULD... contain information about the physical consequences of an eating disorder (e.g. medical complications such as bone density loss or cardiac problems) (12-17) | 90.91 | 85.71 | 100.00 | 80.00 |
| Presentations SHOULD... contain information about the physical consequences of an eating disorder (e.g. medical complications such as bone density loss or cardiac problems) (18+) | 90.91 | 93.33 | 100.00 | 90.00 |
| Presentations SHOULD NOT... mention specific clothing sizes of a person with an eating disorder (12-17) | 85.71 | 90.00 | 85.71 (HARMFUL) | 90.00 (HARMFUL) |
| Presentations SHOULD NOT... mention specific clothing sizes of a person with an eating disorder (18+) | 85.71 | 90.00 | 85.71 (HARMFUL) | 90.00 (HARMFUL) |
| Authors and presenters SHOULD… explain that many people with eating disorders may be of average or above average weight and shape (12-17) | 100.00 | 100.00 | 85.71 | 90.00 |
| Authors and presenters SHOULD... explain the dangers of weight control behaviours (e.g. laxatives, vomiting) (12-17) | 85.71 | 90.00 | 100.00 | 80.00 |
| Authors and presenters SHOULD... explain the dangers of weight control behaviours (e.g. laxatives, vomiting) (18+) | 100.00 | 80.00 | 100.00 | 90.00 |
| Authors and presenters SHOULD... explain the harmful effects of fad diets (18+) | 85.71 | 80.00 | 100.00 | 80.00 |
| Presentations SHOULD… include information about the psychological impact of an eating disorder, (e.g. thoughts being taken over by weight and food, depression, fear) (12-17) | 90.91 | 85.71 | 100.00 | 100.00 |
| Presentations SHOULD NOT... mention specific amounts of laxatives taken by a person with an eating disorder (12-17) | 81.82 | 85.71 | 100.00 (HARMFUL) | 90.00 (HARMFUL) |
| Presentations SHOULD NOT... mention specific amounts of laxatives taken by a person with an eating disorder (18+) | 81.82 | 86.67 | 100.00 (HARMFUL) | 90.00 (HARMFUL) |
| Presentations SHOULD NOT… mention the frequency or intensity of exercise done by a person with an eating disorder (12-17) | 85.71 | 80.00 | 100.00 (HARMFUL) | 90.00 (HARMFUL) |
| Presentations SHOULD NOT… mention the frequency or intensity of exercise done by a person with an eating disorder (18+) | 85.71 | 80.00 | 100.00 (HARMFUL) | 90.00 (HARMFUL) |
| Presentations SHOULD NOT… mention specific methods used in eating disorder behaviour (e.g. using fingers to purge) (12-17) | 90.91 | 85.71 | 100.00 (HARMFUL) | 90.00 (HARMFUL) |
| Presentations SHOULD NOT… mention specific methods used in eating disorder behaviour (e.g. using fingers to purge) (18+) | 85.71 | 90.00 | 100.00 (HARMFUL) | 90.00 (HARMFUL) |
| Presentations SHOULD NOT… teach or promote calorie/kilojoule counting (12-17) | 85.71 | 90.00 | 100.00 (HARMFUL) | 90.00 (HARMFUL) |
| Presentations SHOULD NOT… teach or promote calorie/kilojoule counting (18+) | 85.71 | 90.00 | 100.00 (HARMFUL) | 90.00 (HARMFUL) |
| Authors and presenters SHOULD NOT… portray eating disorders as caused by bad parenting or celebrity culture (12-17) | 100.00 | 84.62 | 85.71 (HARMFUL) | 100.00 (HARMFUL) |
| Authors and presenters SHOULD NOT… portray eating disorders as caused by bad parenting or celebrity culture (18+) | 100.00 | 85.71 | 85.71 (HARMFUL) | 100.00 (HARMFUL) |
| Presentations SHOULD...discuss the potential harm of appearance related teasing (12-17) | 100.00 | 84.62 | 100.00 | 90.00 |
| Presentations SHOULD...discuss the potential harm of appearance related teasing (18+) | 85.71 | 90.00 | 85.71 | 90.00 |
| Authors and presenters SHOULD NOT... oversimplify the causes of eating disorders (12-17) | 100.00 | 92.31 | 100.00 (HARMFUL) | 90.00 (HARMFUL) |
| Authors and presenters SHOULD NOT... oversimplify the causes of eating disorders (18+) | 100.00 | 92.86 | 100.00 (HARMFUL) | 90.00 (HARMFUL) |
| Authors and presenters SHOULD... explain that most people who recover from an eating disorder do so with the support of both trained professionals, family and/or friends, rather than on their own. (12-17) | 100.00 | 80.00 | 100.00 | 80.00 |
| Authors and presenters SHOULD... explain that most people who recover from an eating disorder do so with the support of both trained professionals, family and/or friends, rather than on their own. (18+) | 100.00 | 80.00 | 100.00 | 80.00 |
| Presentations SHOULD… explain that seeking help early can result in better treatment outcomes (12-17) | 81.82 | 84.62 | 100.00 | 100.00 |
| Presentations SHOULD… explain that seeking help early can result in better treatment outcomes (18+) | 81.82 | 85.71 | 100.00 | 100 |

^a^ Speakers with lived experience not endorsed for this age group. ^b^ This item was part of a deconstructed item, rated on helpfulness/harm for a speaker  ^c^ Helpful/harmful scale not included as it was not necessary.

Table 3*. Items endorsed in round three by scale and panel group (% agreement)*

|  | *Should this be included in guidelines?* | | *How helpful/harmful would this be?* | |
| --- | --- | --- | --- | --- |
| Statement | Professionals | Advocates | Professionals | Advocates |
| Authors SHOULD… consider designing the content of a presentation to be non-gender specific (12-17) | 80.00 | 87.50 | 81.82 | 84.62 |
| Authors SHOULD... be aware that if they plan to have a speaker with lived experience, it is best that the speaker be supported (e.g. given guidance for how to tell their story, provided with debriefing following their presentation) by a mental health organisation with experience in providing community education programs about eating disorders (18+) | 100.00 | 100.00 | 100.00 | 100.00 |
| Authors SHOULD… invite a professional to be available for questions that may arise during the discussion period, if someone is presenting a lived experience story (12-17)^a^ | 80.00 | 100.00 | 90.00 | 85.71 |
| Authors SHOULD… invite a professional to be available for questions that may arise during the discussion period, if someone is presenting a lived experience story (18+) | 80.00 | 87.50 | 80.00 | 86.67 |
| LE Speakers being prepared to address common myths about eating disorders is likely to be… (for the speaker)^b^ |  |  | 100.00 | 87.50 |
| Presentations SHOULD NOT... include images of people with extreme weights or shapes (12-17) | 80.00 | 87.50 | 100.00 (HARMFUL) | 90.00 (HARMFUL) |
| Presentations SHOULD... include a Q&A session with mental health professionals if screening a documentary about eating disorders (18+) | 80.00 | 87.50 | 100.00 | 90.00 |
| Authors and presenters SHOULD ...use accurate psychiatric terms for eating disorders (i.e. anorexia nervosa rather than 'extreme dieting') (12-17) | 100.00 | 87.50 | 80.00 | 85.71 |
| Authors and presenters SHOULD ...use accurate psychiatric terms for eating disorders (i.e. anorexia nervosa rather than 'extreme dieting') (18+) | 100.00 | 87.50 | 80.00 | 86.67 |
| Presentations SHOULD…describe the different types of eating disorders that exist (12-17) | 80.00 | 87.50 | 90.00 | 85.71 |
| Presentations SHOULD NOT… include amount of weight lost by a person with an eating disorder (12-17) | 80.00 | 87.50 | 100.00 (HARMFUL) | 80.00 (HARMFUL) |
| Authors and presenters SHOULD... explain the harmful effects of fad diets (12-17) | 100.00 | 87.50 | 100.00 | 87.50 |
| Presentations SHOULD NOT... include the number of calories/kilojoules eaten by a person with an eating disorder (12-17) | 80.00 | 85.71 | 100.00 (HARMFUL) | 100.00 (HARMFUL) |
| Presentations SHOULD NOT... include the number of calories/kilojoules eaten by a person with an eating disorder (18+) | 80.00 | 85.71 | 100.00 (HARMFUL) | 100.00 (HARMFUL) |
| Presentations SHOULD NOT... include quantifiable details of any disordered eating behaviours or symptoms (12-17) | 80.00 | 100.00 | 80.00 (HARMFUL) | 87.50 (HARMFUL) |
| Presentations SHOULD NOT… mention specific steps taken to disguise eating disorder behaviour (e.g. locking door to hide excessive exercise) (12-17) | 81.82 | 85.71 | 80.00 (HARMFUL) | 87.50 (HARMFUL) |
| Presentations SHOULD… discuss the dangers of binge eating behaviours (12-17) | 81.82 | 92.31 | 80.00 | 87.50 |
| Presentations SHOULD… discuss the dangers of binge eating behaviours (18+) | 80.00 | 93.33 | 100.00 | 87.50 |
| Authors and presenters SHOULD… describe protective factors against the development of eating disorders, such as high self-esteem and life skills (12-17) | 85.71 | 90.00 | 100.00 | 87.50 |
| Authors and presenters SHOULD… describe protective factors against the development of eating disorders, such as high self-esteem and life skills (18+) | 85.71 | 90.00 | 100.00 | 87.50 |
| Presentations SHOULD... address how to recognise and prevent relapse (18+) | 80.00 | 100.00 | 100.00 | 100.00 |
| Presentations SHOULD… emphasise that athletes who have symptoms of an eating disorder are at particular risk for undernourishment and dehydration (18+) | 90.91 | 92.86 | 100.00 | 87.50 |
| Presentations SHOULD… explain the consequences of eating disorders on performance, such as loss of muscular strength and endurance, decreased speed, loss of coordination, and poor judgement. (12-17) | 100.00 | 100.00 | 100.00 | 87.50 |
| Presentations SHOULD… explain the consequences of eating disorders on performance, such as loss of muscular strength and endurance, decreased speed, loss of coordination, and poor judgement. (18+) | 100.00 | 100.00 | 100.00 | 87.50 |
| Presentations SHOULD… address the way in which extreme weight control behaviours (e.g. self-induced vomiting, laxative use) can hurt performance (12-17) | 100.00 | 87.50 | 100.00 | 87.50 |
| Presentations SHOULD… address the way in which extreme weight control behaviours (e.g. self-induced vomiting, laxative use) can hurt performance (18+) | 100.00 | 87.50 | 100.00 | 87.50 |

^a^ Speakers with lived experience not endorsed for this age group. ^b^ This item was part of a deconstructed item, rated on helpfulness/harm for a speaker  ^c^ Helpful/harmful scale not included as it was not necessary.

Table 4*. Items not endorsed in round one by scale and panel group (% agreement)*

|  | *Should this be included in guidelines?* | | *How helpful/harmful would this be?* | |
| --- | --- | --- | --- | --- |
| *Statement* | Professionals | Advocates | Professionals | Advocates |
| Presentations SHOULD… be entertaining (e.g. include a drama performance) (12-17) | 40.00 | 28.57 | 50.00 | 42.86 |
| Presentations SHOULD… be entertaining (e.g. include a drama performance) (18+) | 20.00 | 26.67 | 40.00 | 33.33 |
| Authors SHOULD... present to single sex groups (i.e. female-only or male-only audiences) (12-17) | 9.09 | 21.43 | 18.18 | 28.57 |
| Authors SHOULD... present to single sex groups (i.e. female-only or male-only audiences) (18+) | 0.00 | 20.00 | 9.09 | 20.00 |
| Authors SHOULD... present to mixed gender groups (i.e. males and females in the audience) (12-17) | 45.45 | 71.43 | 36.36 | 69.23 |
| Authors SHOULD... present to mixed gender groups (i.e. males and females in the audience) (18+) | 45.45 | 66.67 | 45.45 | 64.29 |
| Authors SHOULD... be aware that females and males may feel uncomfortable discussing body image/eating disorders in front of the opposite gender (18+) | 81.82 | 73.33 | 77.78 | 66.67 |
| Presentations SHOULD NOT... include media reports, drama presentations or case studies about eating disorders, as they may trivialise or glamorise the illness (12-17) | 18.18 | 21.43 | 18.18 | 35.71 |
| Presentations SHOULD NOT... include media reports, drama presentations or case studies about eating disorders, as they may trivialise or glamorise the illness (18+) | 18.18 | 20.00 | 18.18 | 33.33 |
| Presentations SHOULD... include media reports, drama presentations or case studies about eating disorders that do not trivialise or glamorise the illness (e.g. ones that do not feature celebrities) (12-17) | 63.64 | 42.86 | 63.64 | 50.00 |
| Presentations SHOULD... include media reports, drama presentations or case studies about eating disorders that do not trivialise or glamorise the illness (e.g. ones that do not feature celebrities) (18+) | 63.64 | 40.00 | 63.64 | 33.33 |
| If the author wants to target parents, they SHOULD... use activities led by students to increase attendance, rather than information nights (12-17) | 54.55 | 53.33 | 54.55 | 60.00 |
| Authors SHOULD... include an appropriate speaker with lived experience (12-17) | 54.55 | 85.71 | 70.00 | 78.57 |
| Presentations SHOULD NOT... invite speakers with lived experience who are peers known to the audience (12-17) | 36.36 | 28.57 | 10.00 | 21.43 |
| Presentations SHOULD NOT... invite speakers with lived experience who are peers known to the audience (18+) | 27.27 | 26.67 | 10.00 | 20.00 |
| Authors SHOULD... be aware that if they plan to have a LE speaker, it is best that the LE speaker be endorsed by a mental health organisation (18+) | 63.64 | 20.00 | 70.00 | 40.00 |
| Authors and presenters SHOULD NOT... discuss the symptoms and detrimental effects of eating disorders when the intention of the presentation is prevention, as there is not sufficient evidence to suggest that this is effective. (12-17) | 45.45 | 35.71 | 40.00 | 28.57 |
| Authors and presenters SHOULD NOT... discuss the symptoms and detrimental effects of eating disorders when the intention of the presentation is prevention, as there is not sufficient evidence to suggest that this is effective. (18+) | 36.36 | 40.00 | 30.00 | 33.33 |
| Authors and presenters SHOULD… be aware that there is not sufficient evidence to suggest that discussing the causes of eating disorders will help prevent them (12-17) | 63.64 | 78.57 | 77.78 | 71.43 |
| Authors and presenters SHOULD… be aware that there is not sufficient evidence to suggest that discussing the causes of eating disorders will help prevent them (18+) | 63.64 | 80.00 | 77.78 | 66.67 |
| LE Speakers SHOULD... be eating disorder behaviour free for at least 1 year before presenting (12-17) | 72.73 | 42.86 | 80.00 | 35.71 |
| LE Speakers SHOULD... be eating disorder behaviour free for at least 1 year before presenting (18+) | 63.64 | 26.67 | 80.00 | 33.33 |
| LE Speakers SHOULD… consult with their current or past professional to ask for their feedback about their decision to share their story (12-17) | 63.64 | 50.00 | 80.00 | 46.15 |
| LE Speakers SHOULD… consult with their current or past professional to ask for their feedback about their decision to share their story (18+) | 54.55 | 26.67 | 80.00 | 35.71 |
| LE speakers SHOULD... communicate their story and express opinions only from their perspective (12-17) | 72.73 | 35.71 | 70.00 | 35.71 |
| LE speakers SHOULD... communicate their story and express opinions only from their perspective (18+) | 72.73 | 40.00 | 70.00 | 40.00 |
| LE Speakers SHOULD NOT… speak on behalf of others with lived experience (12-17) | 63.64 | 64.29 | 70.00 | 50.00 |
| LE Speakers SHOULD NOT… speak on behalf of others with lived experience (18+) | 63.64 | 60.00 | 70.00 | 46.67 |
| LE speakers SHOULD...  talk about what their feelings of self-worth and self-esteem were like during their illness (12-17) | 45.45 | 42.86 | 60.00 | 50.00 |
| LE speakers SHOULD...  talk about what their feelings of self-worth and self-esteem were like during their illness (18+) | 45.45 | 60.00 | 60.00 | 53.33 |
| LE speakers SHOULD… discuss the emotions they felt during the times they struggled, (e.g. anger, sadness, shame, fear) (12-17) | 70.00 | 57.14 | 80.00 | 57.14 |
| LE speakers SHOULD… discuss the emotions they felt during the times they struggled, (e.g. anger, sadness, shame, fear) (18+) | 63.64 | 66.67 | 80.00 | 60.00 |
| LE speakers SHOULD NOT… discuss specific details of substance abuse (e.g. amounts or types of drugs taken) (12-17) | 54.55 | 50.00 | 18.18 | 42.86 |
| LE speakers SHOULD NOT… discuss specific details of substance abuse (e.g. amounts or types of drugs taken) (18+) | 54.55 | 60.00 | 18.18 | 53.33 |
| LE Speakers SHOULD NOT... specify the frequency or duration  they engaged in eating disorder behaviours (12-17) | 54.55 | 64.29 | 10.00 | 50.00 |
| LE Speakers SHOULD NOT... specify the frequency or duration  they engaged in eating disorder behaviours (18+) | 54.55 | 60.00 | 10.00 | 46.67 |
| LE speakers SHOULD NOT... make reference to specific treatment facilities or providers (12-17) | 36.36 | 21.43 | 20.00 | 28.57 |
| LE speakers SHOULD NOT... make reference to specific treatment facilities or providers (18+) | 36.36 | 20.00 | 20.00 | 20.00 |
| LE speakers SHOULD... talk about the experience of going into hospital if relevant to their story (12-17) | 54.55 | 28.57 | 70.00 | 42.86 |
| LE speakers SHOULD... talk about the experience of going into hospital if relevant to their story (18+) | 54.55 | 40.00 | 70.00 | 53.33 |
| Presentations SHOULD NOT… include images of people with extreme weights or shapes (18+) | 54.55 | 66.67 | 20.00 | 40.00 |
| Presentations SHOULD… include images that promote shape and size diversity (12-17) | 81.82 | 64.29 | 70.00 | 64.29 |
| Presentations SHOULD… include images that promote shape and size diversity (18+) | 81.82 | 73.33 | 70.00 | 66.67 |
| Presentations SHOULD NOT... mention pro-eating disorder websites (12-17) | 45.45 | 35.71 | 30.00 | 28.57 |
| Presentations SHOULD NOT... mention pro-eating disorder websites (18+) | 27.27 | 26.67 | 10.00 | 26.67 |
| Presentations SHOULD NOT... include movies depicting persons with eating disorders (12-17) | 27.27 | 35.71 | 10.00 | 35.71 |
| Presentations SHOULD NOT... include movies depicting persons with eating disorders (18+) | 18.18 | 33.33 | 10.00 | 26.67 |
| Authors SHOULD… consider showing videos that promote body image media literacy (18+) | 72.73 | 53.33 | 90.00 | 60.00 |
| Presentations SHOULD NOT... Include images or descriptions of a person's appearance during periods of severe eating disorder symptoms (18+) | 63.64 | 73.33 | 30.00 | 40.00 |
| Authors SHOULD NOT... include portrayals of people with eating disorders that elicit fear (12-17) | 63.64 | 71.43 | 20.00 | 42.86 |
| Authors SHOULD NOT... include portrayals of people with eating disorders that elicit fear (18+) | 54.55 | 66.67 | 20.00 | 46.67 |
| Authors SHOULD… consider screening a documentary about eating disorders (12-17) | 36.36 | 21.43 | 40.00 | 35.71 |
| Authors SHOULD… consider screening a documentary about eating disorders (18+) | 36.36 | 40.00 | 50.00 | 53.33 |
| Presentations SHOULD... include a Q&A session with mental health professionals if screening a documentary about eating disorders (12-17) | 81.82 | 64.29 | 60.00 | 71.43 |
| Presentations SHOULD... provide the audience with role models who are praised for their achievements and personal qualities rather than their appearance (12-17) | 63.64 | 71.43 | 80.00 | 78.57 |
| Presentations SHOULD... provide the audience with role models who are praised for their achievements and personal qualities rather than their appearance (18+) | 54.55 | 73.33 | 80.00 | 73.33 |
| Authors and presenters SHOULD… use language that presents people suffering eating disorders as experiencing immense distress (12-17) | 72.73 | 57.14 | 80.00 | 42.86 |
| Authors and presenters SHOULD… use language that presents people suffering eating disorders as experiencing immense distress (18+) | 72.73 | 60.00 | 80.00 | 46.67 |
| Presentations SHOULD… advocate for treatment and research in eating disorders (12-17) | 81.82 | 64.29 | 70.00 | 57.14 |
| Presentations SHOULD NOT… discuss the number of hospital admissions of a person with an eating disorder (12-17) | 36.36 | 71.43 | 10.00 | 35.71 |
| Presentations SHOULD NOT… discuss the number of hospital admissions of a person with an eating disorder (18+) | 36.36 | 60.00 | 10.00 | 33.33 |
| Authors and presenters SHOULD… explain the harmful effects of steroid use (12-17) | 72.73 | 50.00 | 77.78 | 42.86 |
| Authors and presenters SHOULD… explain the harmful effects of steroid use (18+) | 72.73 | 53.33 | 80.00 | 46.67 |
| Presentations SHOULD… discuss the way in which friends and family can be negatively impacted by an eating disorder (12-17) | 45.45 | 78.57 | 80.00 | 57.14 |
| Presentations SHOULD… discuss the way in which friends and family can be negatively impacted by an eating disorder (18+) | 63.64 | 93.33 | 90.00 | 66.67 |
| Presentations SHOULD NOT… mention unhealthy methods of weight control (e.g. diet pills, smoking) (12-17) | 60.00 | 69.23 | 22.22 | 38.46 |
| Presentations SHOULD NOT… mention unhealthy methods of weight control (e.g. diet pills, smoking) (18+) | 40.00 | 53.33 | 11.11 | 26.67 |
| Presentations SHOULD NOT… teach or promote reading food labels (12-17) | 63.64 | 64.29 | 20.00 | 42.86 |
| Presentations SHOULD NOT… teach or promote reading food labels (18+) | 63.64 | 66.67 | 20.00 | 40.00 |
| Authors and presenters SHOULD NOT… describe eating disorders as caused by biogenetic/genetic factors because this may reinforce the idea that an eating disorder is an unchangeable characteristic of a person. (12-17) | 20.00 | 30.77 | 22.22 | 23.08 |
| Authors and presenters SHOULD NOT… describe eating disorders as caused by biogenetic/genetic factors because this may reinforce the idea that an eating disorder is an unchangeable characteristic of a person. (18+) | 20.00 | 21.43 | 22.22 | 21.43 |
| Presentations SHOULD NOT… suggest that psychosocial factors are more controllable than biogenetic factors in the development of an eating disorder (12-17) | 30.00 | 38.46 | 22.22 | 30.77 |
| Presentations SHOULD NOT… suggest that psychosocial factors are more controllable than biogenetic factors in the development of an eating disorder (18+) | 30.00 | 35.71 | 25.00 | 28.57 |
| Presentations SHOULD... describe eating disorders as caused by biogenetic risk factors because this may reduce personal blame for the development of an eating disorder. (12-17) | 30.00 | 30.77 | 33.33 | 30.77 |
| Presentations SHOULD... describe eating disorders as caused by biogenetic risk factors because this may reduce personal blame for the development of an eating disorder. (18+) | 30.00 | 35.71 | 33.33 | 35.71 |
| Authors and presenters SHOULD…explain that holding people responsible for their eating disorder behaviour may assist people in their efforts to recover (12-17) | 30.00 | 15.38 | 33.33 | 15.38 |
| Authors and presenters SHOULD…explain that holding people responsible for their eating disorder behaviour may assist people in their efforts to recover (18+) | 30.00 | 21.43 | 33.33 | 21.43 |
| Authors and presenters SHOULD NOT… use stories of people who fought their illness alone (12-17) | 36.36 | 15.38 | 30.00 | 15.38 |
| Authors and presenters SHOULD NOT… use stories of people who fought their illness alone (18+) | 18.18 | 0.00 | 11.11 | 7.69 |
| Authors and presenters SHOULD… explain that treatment for Bulimia Nervosa does not typically result in weight gain (12-17) | 54.55 | 69.23 | 55.56 | 61.54 |
| Authors and presenters SHOULD… explain that treatment for Bulimia Nervosa does not typically result in weight gain (18+) | 54.55 | 71.43 | 60.00 | 64.29 |
| Presentations SHOULD NOT… include specific names of psychiatric medication (12-17) | 45.45 | 46.15 | 40.00 | 23.08 |
| Presentations SHOULD NOT… include specific names of psychiatric medication (18+) | 36.36 | 42.86 | 40.00 | 23.08 |
| Presentations SHOULD… explain that psychiatric medication may be beneficial for someone with eating disorders (12-17) | 63.64 | 61.54 | 70.00 | 53.85 |
| Presentations SHOULD… explain that psychiatric medication may be beneficial for someone with eating disorders (18+) | 63.64 | 64.29 | 70.00 | 57.14 |
| Presentations SHOULD NOT… include specific doses of psychiatric medication (12-17) | 63.64 | 61.54 | 50.00 | 23.08 |
| Presentations SHOULD NOT… include specific doses of psychiatric medication (18+) | 63.64 | 57.14 | 55.56 | 21.43 |
| Presentations SHOULD NOT... reference specific treatment facilities or providers (12-17) | 9.09 | 7.69 | 30.00 | 0.00 |
| Presentations SHOULD NOT... reference specific treatment facilities or providers (18+) | 9.09 | 7.69 | 30.00 | 0.00 |
| Presenters SHOULD... encourage audience members who think they or a friend have an eating disorder to speak to the presenter or another person (12-17) | 45.45 | 76.92 | 50.00 | 69.23 |
| Presenters SHOULD... encourage audience members who think they or a friend have an eating disorder to speak to the presenter or another person (18+) | 36.36 | 71.43 | 40.00 | 64.29 |
| Presenters SHOULD… suggest ways to engage in advocacy activities (12-17) | 18.18 | 53.85 | 30.00 | 46.15 |
| Presenters SHOULD… suggest ways to engage in advocacy activities (18+) | 18.18 | 57.14 | 30.00 | 50.00 |
| Presenters SHOULD... explore social perceptions of people with eating disorders (12-17) | 54.55 | 61.54 | 60.00 | 53.85 |
| Presenters SHOULD... explore social perceptions of people with eating disorders (18+) | 54.55 | 64.29 | 60.00 | 57.14 |
| Presenters SHOULD... provide a definition of mental illness and mental health (12-17) | 27.27 | 69.23 | 20.00 | 69.23 |
| Presenters SHOULD... provide a definition of mental illness and mental health (18+) | 27.27 | 71.43 | 20.00 | 71.43 |
| Presentations about eating disorders SHOULD NOT... be given until high school (children aged 12 and up) (under 12) | 27.27 | 14.29 | 30.00 | 7.14 |
| Presentations to children under 12 SHOULD... focus on prevention only and not mention eating disorders (under 12) | 54.55 | 28.57 | 66.67 | 28.57 |
| Presentations SHOULD… address the importance of seeking help for menstrual irregularities at the first sign of abnormality as this can be a symptom of an eating disorder (12-17) | 72.73 | 38.46 | 70.00 | 30.77 |
| Presentations SHOULD… address the importance of seeking help for menstrual irregularities at the first sign of abnormality as this can be a symptom of an eating disorder (18+) | 63.64 | 50.00 | 70.00 | 42.86 |
| Presentations SHOULD... include nutrition experts to educate about optimal eating for performance (12-17) | 45.45 | 46.15 | 50.00 | 46.15 |
| Presentations SHOULD... include nutrition experts to educate about optimal eating for performance (18+) | 45.45 | 57.14 | 50.00 | 50.00 |
| Authors SHOULD… be aware that information about the causes, symptoms, and detrimental effects of eating disorders should only be presented to persons who care for people at risk (e.g. parents, staff, coaches) (12-17) | 27.27 | 15.38 | 20.00 | 15.38 |
| Authors SHOULD… be aware that information about the causes, symptoms, and detrimental effects of eating disorders should only be presented to persons who care for people at risk (e.g. parents, staff, coaches) (18+) | 18.18 | 21.43 | 20.00 | 21.43 |
| Authors SHOULD... be aware that talking about eating disorder behaviours is potentially harmful to children (12-17) | 72.73 | 69.23 | 50.00 | 46.15 |

^a^ Speakers with lived experience not endorsed for this age group. ^b^ This item was part of a deconstructed item, rated on helpfulness/harm for a speaker  ^c^ Helpful/harmful scale not included as it was not necessary.

Table 5*. Items not endorsed in round two by scale and panel group (% agreement)*

|  | *Should this be included in guidelines?* | | *How helpful/harmful would this be?* | |
| --- | --- | --- | --- | --- |
| *Statement* | Professionals | Advocates | Professionals | Advocates |
| Presentations SHOULD… present the information in an interactive and participatory way (e.g. group discussion, group activities and peer based learning) (18+) | 71.43 | 40.00 | 90.91 | 60.00 |
| Authors SHOULD… provide presentations about body image and self-esteem within their presentation on eating disorders (18+) | 42.86 | 60.00 | 100.00 | 80.00 |
| Authors SHOULD... be aware that single session presentations are less effective than multi-session presentations (12-17) | 71.43 | 60.00 | 80.00 | 42.86 |
| Authors SHOULD... be aware that single session presentations are less effective than multi-session presentations (18+) | 71.43 | 50.00 | 80.00 | 46.67 |
| Authors SHOULD… consider the needs of people for whom English is a second language when designing a presentation (12-17) | 42.86 | 60.00 | 85.71 | 80.00 |
| Authors SHOULD… consider the needs of people for whom English is a second language when designing a presentation (18+) | 42.86 | 60.00 | 85.71 | 80.00 |
| Authors SHOULD... include an appropriate speaker with lived experience (18+) | 28.57 | 70.00 | 70.00 | 93.33 |
| Presenters with lived experience who do not have a supporting organisation experienced in providing community education programs about eating disorders SHOULD NOT... attempt presentations on their own. (18+) | 28.57 | 60.00 | 57.14 (HARMFUL) | 70.00 (HARMFUL) |
| Authors and presenters SHOULD... be aware that if their purpose is to PREVENT eating disorders, they should use an existing, evidence-based, evaluated program, rather than design their own (18+) | 71.43 | 80.00 | 57.14 (HARMFUL) | 70.00 (HARMFUL) |
| Speakers with lived experience SHOULD... be eating disorder behaviour free for more than one year before presenting (18+) | 50.00 | 57.14 | 100.00 | 80.00 |
| Presenters with lived experience who do not have a supporting organisation experienced in providing community education programs about eating disorders SHOULD NOT... attempt presentations on their own. (12-17) | 42.85 | 60.00 | 57.14 (HARMFUL) | 60.00 (HARMFUL) |
| Presenters with lived experience who do not have a supporting organisation experienced in providing community education programs about eating disorders SHOULD NOT... attempt presentations on their own. (18+) | 28.57 | 60.00 | 57.14 (HARMFUL) | 70.00 (HARMFUL) |
| Speakers with lived experience SHOULD... be aware that attempting to give a presentation without the support of an organisation experienced in providing community education programs about eating disorders is likely to be… (for the speaker)^b^ |  |  | 57.14 (HARMFUL) | 60.00 (HARMFUL) |
| People with lived experience who wish to share their story to aid their own recovery SHOULD…consider becoming involved in a peer support network rather than providing community presentations (12-17) | 71.43 | 60.00 | 28.57 | 60.00 |
| People with lived experience who wish to share their story to aid their own recovery SHOULD…consider becoming involved in a peer support network rather than providing community presentations (18+) | 71.43 | 60.00 | 28.57 | 50.00 |
| Presentations to children under 12 SHOULD... only occur if eating disorders have become relevant in a child’s social network (i.e. a child at school has developed a disorder) (Rated for children under 12) | 57.14 | 40.00 | 71.43 | 60.00 |
| Presentations to children under 12 SHOULD only occur if eating disorders have become relevant in a child’s social network (i.e. a child at school has developed a disorder) and should focus on myth-busting (Rated for children under 12) | 42.86 | 30.00 | 71.43 | 90.00 |
| Presentations to children under 12 SHOULD only occur if eating disorders have become relevant in a child’s social network (i.e. a child at school has developed a disorder) and should focus on preventing other children from engaging in disordered eating behaviours (Rated for children under 12) | 57.14 | 50.00 | 71.43 | 80.00 |
| If eating disorders have become relevant in the social network of a child who is 12 or under (e.g. a child at school has developed a disorder), presentations about eating disorders SHOULD only be given to parents and/or teachers (Rated for children under 12) | 57.14 | 30.00 | 71.43 | 40.00 |
| Presentations SHOULD… emphasise that athletes who have symptoms of an eating disorder are at particular risk for undernourishment and dehydration (12-17) | 71.43 | 60.00 | 85.71 | 70.00 |

^a^ Speakers with lived experience not endorsed for this age group. ^b^ This item was part of a deconstructed item, rated on helpfulness/harm for a speaker  ^c^ Helpful/harmful scale not included as it was not necessary.

Table 6*. Items not endorsed in round three by scale and panel group (% agreement)*

|  | *Should this be included in guidelines?* | | *How helpful/harmful would this be?* | |
| --- | --- | --- | --- | --- |
| *Statement* | Professionals | Advocates | Professionals | Advocates |
| Presentations SHOULD… present the information in an interactive and participatory way (e.g. group discussion, group activities and peer based learning) (12-17) | 80.00 | 62.50 | 100.00 | 64.29 |
| Authors SHOULD… consider designing the content of a presentation to be non-gender specific (18+) | 80.00 | 87.50 | 72.73 | 85.71 |
| Authors SHOULD… provide presentations about body image and self-esteem in addition to eating disorders (18+) | 80.00 | 75.00 | 100.00 | 80.00 |
| Authors SHOULD... consider using an appropriate speaker with lived experience (12-17) | 40.00 | 75.00 | 100.00 | 90.00 |
| Presentations SHOULD… only discuss quantifiable details of eating disorder symptoms and behaviours (e.g. BMI, calories eaten, frequency or intensity of exercise) when the audience is a professional one (e.g. medical doctors, clinical psychologists, professionals in training) (for a professional audience) | 80.00 | 63.00 | 80.00 | 37.50 |
| Authors and presenters SHOULD... be aware that if their purpose is to PREVENT eating disorders, they should use an existing, evidence-based, evaluated program, rather than design their own (12-17) | 100.00 | 75.00 | 100.00 (HARMFUL) | 87.50 (HARMFUL) |
| Authors and presenters SHOULD... be aware that if their purpose is to PREVENT eating disorders, they should use an existing, evidence-based, evaluated program, rather than design their own (18+) | 100.00 | 75.00 | 57.14 (HARMFUL) | 70.00 (HARMFUL) |
| LE speakers wanting to share their story SHOULD... first seek support from a mental health organisation with experience in providing community education programs about eating disorders (12-17)^a^ | 100.00 | 75.00 | 100.00 | 100.00 |
| LE speakers wanting to share their story SHOULD... first seek support from a mental health organisation with experience in providing community education programs about eating disorders (18+) | 80.00 | 62.50 | 100.00 | 100.00 |
| Speakers with lived experience SHOULD... be eating disorder behaviour free for more than one year before presenting (12-17)^a^ | 100.00 | 75.00 | 100.00 | 90.00 |
| Speakers with lived experience SHOULD... be eating disorder behaviour free for more than one year before presenting (18+) | 100.00 | 62.50 | 100.00 | 80.00 |
| Speakers with lived experience SHOULD... be eating disorder behaviour free for at least six months before presenting (12-17) ^a^ | 100.00 | 75.00 | 100.00 | 80.00 |
| Speakers with lived experience SHOULD... be eating disorder behaviour free for at least six months before presenting (18+) | 100.00 | 62.50 | 100.00 | 80.00 |
| LE Speakers being prepared to address common myths about eating disorders is likely to be… (for the speaker) |  |  | 100.00 | 87.50 |
| LE Speakers SHOULD... talk about the people who helped move them towards recovery (for adolescents) ^a^ | 100.00 | 75.00 | 80.00 | 87.50 |
| LE Speakers SHOULD... talk about what their loved ones did to help them through recovery (12-17) | 40.00 | 62.50 | 100.00 | 75.00 |
| LE Speakers SHOULD... talk about what their loved ones did to help them through recovery (18+) | 60.00 | 50.00 | 100.00 | 75.00 |
| Presentations SHOULD NOT... include images of people before and after recovery from an eating disorder (18+) | 60.00 | 87.50 | 85.71 (HARMFUL) | 80.00 (HARMFUL) |
| Authors SHOULD consider showing videos that promote body image media literacy (12-17) | 80.00 | 75.00 | 100.00 | 75.00 |
| Presentations SHOULD NOT... Include images or descriptions of a person's appearance during periods of severe eating disorder symptoms (12-17) | 60.00 | 87.50 | 80.00 (HARMFUL) | 62.50 (HARMFUL) |
| Authors and presenters SHOULD NOT... imply that all types of eating disorders are the same (12-17) | 60.00 | 87.50 | 100.00 (HARMFUL) | 85.71 (HARMFUL) |
| Authors and presenters SHOULD… use language that does not define a person by their illness (i.e. 'person with bulimia') rather than language that does (i.e. 'they are bulimic'), unless an LE speaker prefers otherwise. (12-17) | 100.00 | 62.50 | 80.00 (HARMFUL) | 50.00 (HARMFUL) |
| Authors and presenters SHOULD… use language that does not define a person by their illness (i.e. 'person with bulimia') rather than language that does (i.e. 'they are bulimic'), unless an LE speaker prefers otherwise. (18+) | 100.00 | 62.50 | 80.00 (HARMFUL) | 50.00 (HARMFUL) |
| Authors and presenters SHOULD NOT… use language that trivialises eating disorders, i.e. adding the suffix “orexia” to indicate an eating disorder (bigorexia, manorexia) (12-17) | 100.00 | 87.50 | 60.00 (HARMFUL) | 87.50 (HARMFUL) |
| Authors and presenters SHOULD NOT… use language that trivialises eating disorders, i.e. adding the suffix “orexia” to indicate an eating disorder (bigorexia, manorexia) (18+) | 100.00 | 87.50 | 60.00 (HARMFUL) | 75.00 (HARMFUL) |
| Presentations SHOULD… advocate for treatment and research in eating disorders (18+) | 81.82 | 80.00 | 100.00 | 75.00 |
| Authors and presenters SHOULD explain the effects of dieting on body weight regulation (12-17) | 80.00 | 50.00 | 80.00 | 50.00 |
| Authors and presenters SHOULD explain the effects of dieting on body weight regulation (18+) | 80.00 | 75.00 | 80.00 | 62.50 |
| Presentations SHOULD explain that dieting often causes weight gain (12-17) | 100.00 | 50.00 | 100.00 | 50.00 |
| Presentations SHOULD explain that dieting often causes weight gain (18+) | 100.00 | 50.00 | 100.00 | 62.50 |
| Presentations SHOULD NOT... include quantifiable details of any disordered eating behaviours or symptoms (18+) | 60.00 | 100.00 | 60.00 (HARMFUL) | 87.50 (HARMFUL) |
| Presentations SHOULD NOT… mention frequency of eating disorder behaviours (e.g. purging three times a day) (12-17) | 60.00 | 87.50 | 80.00 (HARMFUL) | 87.50 (HARMFUL) |
| Presentations SHOULD NOT… mention frequency of eating disorder behaviours (e.g. purging three times a day) (18+) | 60.00 | 87.50 | 70.00 (HARMFUL) | 57.14 (HARMFUL) |
| Presentations SHOULD NOT… mention specific steps taken to disguise eating disorder behaviour (e.g. locking door to hide excessive exercise) (18+) | 60.00 | 62.50 | 60.00 (HARMFUL) | 75.00 (HARMFUL) |
| Presentations SHOULD... address how to recognise and prevent relapse (12-17) | 60.00 | 100.00 | 90.00 | 85.71 |

^a^ Speakers with lived experience not endorsed for this age group. ^b^ This item was part of a deconstructed item, rated on helpfulness/harm for a speaker  ^c^ Helpful/harmful scale not included as it was not necessary.
